# Gut Microbiota Regulates Systemic Inflammatory Response and Compensatory Anti‐Inflammatory Response Syndromes by Targeting PF4^+^ Macrophages in Acute Pancreatitis

**DOI:** 10.1002/advs.202511193

**Published:** 2026-05-26

**Authors:** Liwei Liu, Guanqun Li, Dongxu Lu, Haoran Ding, Tianqi Lu, Yuhang Sui, Can Zhang, Yu Xie, Rui Kong, Hua Chen, Xuewei Bai, Hongtao Tan, Dongbo Xue, Xianzhi Meng, Le Li, Bei Sun

**Affiliations:** ^1^ Department of Minimally Invasive Biliary Surgery The First Affiliated Hospital of Harbin Medical University Harbin China; ^2^ Key Laboratory of Hepatosplenic Surgery Ministry of Education Harbin China; ^3^ Department of Pancreatic and Biliary Surgery The First Affiliated Hospital of Harbin Medical University Harbin China

**Keywords:** 1‐methylnicotinamide, acute pancreatitis, bacteroides thetaiotaomicron, compensatory anti‐inflammatory response, gut microbiota, PF4^+^ macrophages, systemic inflammatory response

## Abstract

Acute pancreatitis (AP) begins with pancreatic local inflammation, leading to the onset of systemic inflammatory response syndrome (SIRS), followed by compensatory anti‐inflammatory response syndrome (CARS), which causes immune paralysis and higher mortality rate. We have demonstrated that AP disrupts the balance of the gut microbiota that aggravates disease progression; however, the role of gut microbiota in the development of SIRS/CARS remains poorly understood. Here, we observed a lower abundance of *Bacteroides thetaiotaomicron* (*B. thetaiotaomicron*) that increased the infiltration of PF4^+^ macrophages in AP patient and mouse models, which, in turn, promoted the recruitment of Th2 cells and neutrophils and exacerbated SIRS/CARS. Supplementation with *B. thetaiotaomicron* increased the expression of the enzyme N‐methyltransferase (NMMT) and enhanced the production of 1‐methylnicotinamide (1MNA) in the gut epithelial cells, which inhibited PF4^+^ macrophages dependent SIRS/CARS by targeting ELF4, a transcript factor of PF4. Our findings provide novel interventions for AP patients with SIRS/CARS through modulating gut microbiota.

## Graphical abstract

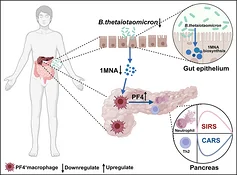

## 1 Introduction

Acute pancreatitis (AP) is an inflammatory process of the pancreas characterized by both local pancreatic injury and systemic inflammatory response [1]. The incidence of AP is estimated at 110 to 140 per 100 000 population, with a total mortality rate of 5%, which can be as high as 20% in severe cases [2, 3]. In AP, the inflammatory mediators released by pancreatic acinar cells, in response to damage, cause pancreatic immune cells infiltration such as neutrophils, macrophages and T cells. In the early phase of AP, the excessive secretion of pro‐inflammatory cytokine such as IL‐6, IL‐1β and TNF‐α cause immune imbalance (increased T cells and suppressed MDSCs) and lead to severe complications, including multiple organ dysfunction syndrome (MODS) and systemic inflammatory response‐syndrome (SIRS) [4, 5]. Compensatory anti‐inflammatory response syndrome (CARS) is a consequence of SIRS to avoid an overactivated inflammatory state which is characterized by activated MDSCs and Tregs that trigger immunosuppressive phenotype and the release of anti‐inflammatory cytokines, such as IL‐10 and IL‐4 [6]. The initiation of CARS is always overlapping with SIRS, which makes it difficult to be diagnosed in the early phase that causes a higher mortality rate [6, 7]. Thus, understanding the immune perturbations‐associated SIRS/CARS phenotype are crucial for improving clinical outcome of AP patients.

Dysbiosis of gut microbiota, commonly described as an imbalance between beneficial and harmful microorganisms, disrupts host immune homeostasis [8, 9, 10]. The gut microbiota disorders increase intestinal barrier permeability which support the microbial metabolites or vesicles released by bacteria enter the circulation that influence local and systemic immune activation [11, 12, 13, 14, 15, 16]. Gut microbiota and host metabolism synergistically control innate and adaptive immune response in the infectious diseases, fibrotic diseases and malignant tumors of the pancreas [9, 17, 18]. Our previous findings have demonstrated that the gut microbiota are involved in regulating innate immune‐mediated AP aggravation by targeting neutrophils extracellular traps or the polarization of macrophages [8, 9, 19], while its role in the adaptive immune response and modulating SIRS/CARS phenotype remains unclear. Uncovering the phenomenon by which gut microbiota modulates innate/adaptive immune cross‐talks in the development of SIRS/CARS and its potential metabolic mechanism can contribute to discover targets for preventing the incidence of severe complications in AP.

In this study, we found that the abundance of *B.thetaiotaomicron* was downregulated in the gut of AP patients, and the clinical relevance between downregulated *B.thetaiotaomicron* with the severity of disease and the incidence of complications were built. We then explored the immune profiles altered by *B.thetaiotaomicron*, and the causal connection between *B.thetaiotaomicron* associated PF4^+^ macrophages with neutrophils/Th2 cells dependent SIRS/CARS was established. Our study demonstrated the metabolic mechanism by which *B.thetaiotaomicron* enhances the production of 1MNA, which alleviates SIRS/CARS in AP by targeting PF4^+^ macrophages. These findings highlight the importance of gut microbiota‐host metabolism interplay in modulating the immune imbalance‐mediated SIRS/CARS phenotype and offers potential therapeutic value of overcoming AP deterioration.

## 2 Results

### 2.1 Gut Microbiota Dysbiosis is Associated With Immune Dysfunction in AP Patients With SIRS/CARS Phenotype

**FIGURE 1:**
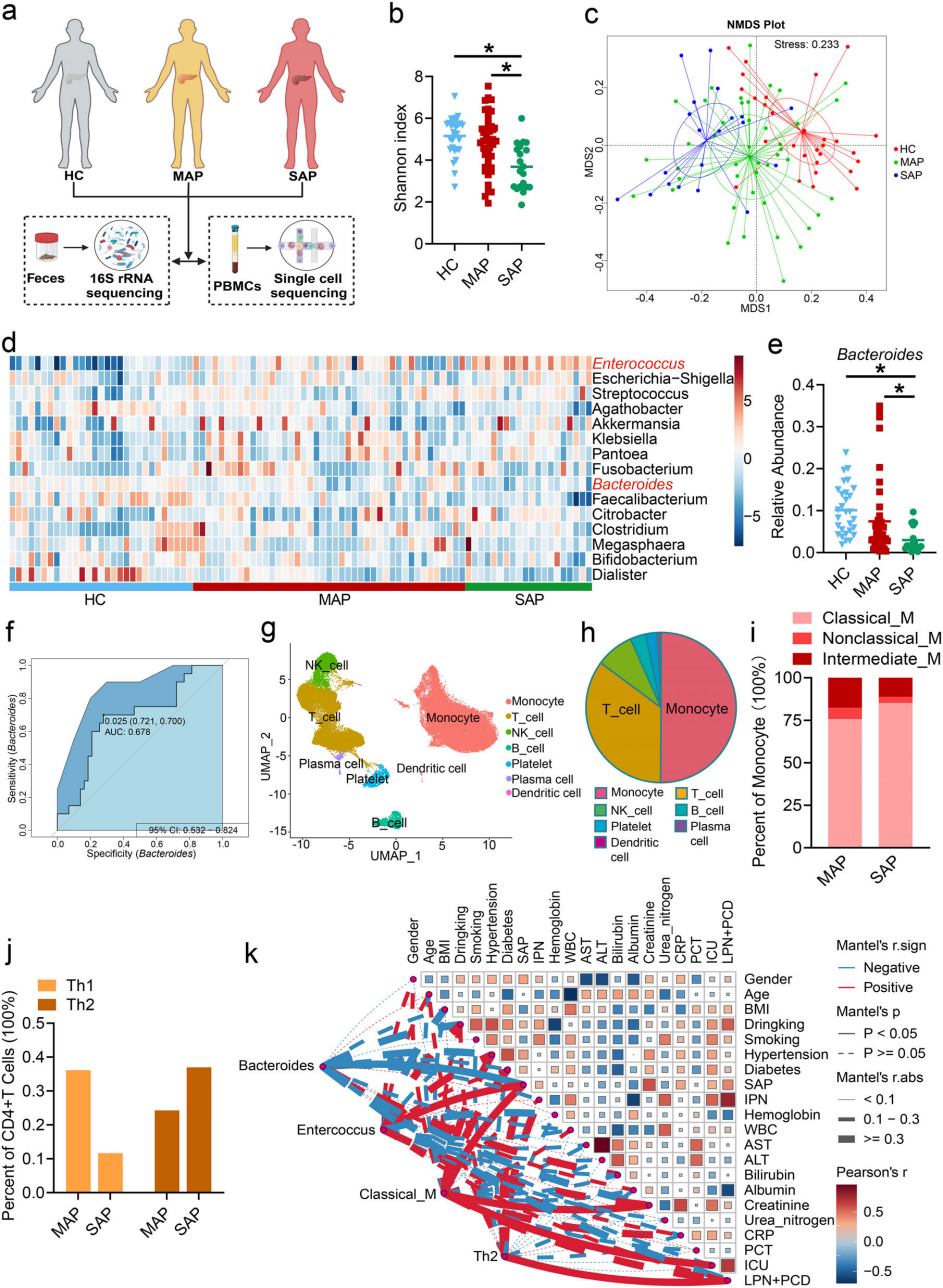
Alternation of gut microbiota and circulating immune landscape in AP patients. (a) Schematic diagram of human research design (healthy controls, n = 29; mild acute pancreatitis patients, n = 43; severe acute pancreatitis patients, n = 20). (b) Alpha diversity index (based on Shannon). (c) Nonmetric multidimensional scaling (NMDS) plot clustering differential microbial distributions between groups. (d) Relative abundance of the top 15 most abundant genera in each sample. (e) Relative abundance of the genera *Bacteroides* in each sample. (f) The ROC curve discriminating SAP vs MAP patients using the abundance of *Bacteroides*. (g) A UMAP displaying distinct clusters of PBMCs in AP patients. (h) Proportion of each cell cluster in human peripheral blood mononuclear cell (hPBMCs). (i) The proportion of the monocytes. (j) The proportion of the CD4+ T cells. (k) Correlation analysis among *Bacteroides, Enterococcus*, classical monocytes, Th2 cells and clinical index. *P* values were determined by two‐tailed ordinary one‐way ANOVA with the Tukey post hoc test or Student's *t*‐test. Data was represented as mean ± SEM. *p < 0.05.

To investigate the association between alternation of gut microbiota and disease severity in AP patients, fecal samples from healthy controls (HC, n = 29) and AP patients (mild acute pancreatitis patients, MAP, n = 43 and severe acute pancreatitis patients, SAP, n = 20) were collected and sequenced (Figure 1a). No differences of baseline information were found within AP subsets (Table S1). The α‐diversity of gut microbiota was decreased in AP patients, particularly in SAP patients compared to HC individuals (Figure 1b). The non‐metric multidimensional scaling (NMDS) analysis indicated significant compositional differences between HC, MAP and SAP (Figure 1c). We then compared the relative abundance of phylum in the gut microbiota and found a decrease in *Bacteroidota*, an increase in *Proteobacteria* in AP patients compared with that in HC individuals (Figure S1a). At the genus level, the abundance of *Enterococcus* was upregulated in AP patients, while *Bacteroides* was downregulated in AP patients compared with HC (Figure 1d, e, Figure S1b‐d). The difference of *Bacteroides* between MAP and SAP was much striking than that in *Enterococcus* (Figure 1e, Figure S1d), and low abundance of *Bacteroides* was associated with increased incidence of SAP (Figure 1f,k, Figure S1d).

Next, we collected the PBMCs of AP patients and the immune profiles was investigated using single cell transcriptomic sequencing (scRNA‐seq). Seven main cell types were annotated and monocyte and T cell accounted for the top two populations. (Figure 1g, h, Figure S1f). The proportion of classical monocytes (CD14^+^ CD16^−^) and Th2 cells (GATA3^+^ CD4^+^) were upregulated in SAP patients, while the proportion of nonclassical monocytes (CD14^−^ CD16^+^), intermediate monocytes (CD14^+^ CD16^+^), CD8^+^ T cells (CD8a^+^ CD3^+^) and Th1 cells (Tbet^+^ CD4^+^) were upregulated in Non‐SAP patients, with no difference in CD4^+^ T cells (CD3^+^ CD4^+^) (Figure 1i,j, Figure S1e). These changes were then confirmed by qPCR (Figure S1g‐i). Classical monocytes and Th2 cells can activate pro‐inflammatory and anti‐inflammatory pathways in AP [5, 6], we then correlated the proportion of both populations with clinical features, and found that the frequency of classical monocytes was positively correlated with ICU admission and acute kidney injury, while the frequency of Th2 cells was positively associated with pancreatic infectious necrosis (Figure S1j, Figure 1k). We then explored the associations between changes of gut microbiota and immune status and found an inverse correlation between the abundance of *Bacteroides* and the frequency of classical monocytes and Th2 cells (Figure S1k, l). These suggest that the interplay between gut microbiota and specific immune subtypes may contribute to pro‐inflammatory‐mediated SIRS and anti‐inflammatory‐mediated CARS during AP.

### 2.2 Gut Microbiota Modulates PF4^+^ Macrophages‐Mediated SIRS/CARS in AP

**FIGURE 2:**
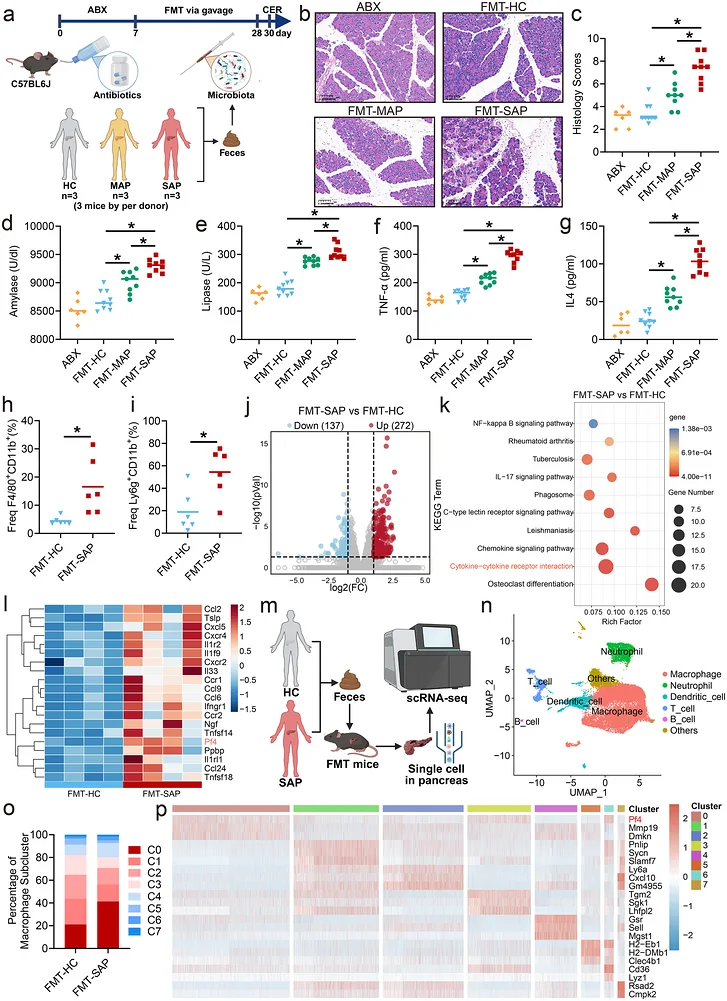
Gut microbiota dysbiosis exacerbates PF4^+^ macrophages‐mediated SIRS/CARS phenotype during AP. (a) Schematic representation of fecal microbiota transplantation (FMT) treatment during AP. C57BL/6 mice were colonized with gut microbiota obtained from HC, MAP and SAP donors (n = 3). The FMT program involves three mice corresponding to one donor. (b) Representative images of hematoxylin and eosin (H&E) staining in the pancreas of FMT‐treated mice (scale bar = 100 µm; ABX, n = 6; FMT‐HC, n = 9; FMT‐MAP, n = 9; FMT‐SAP, n = 9). (c) Quantification of histology score of pancreatic tissues. (d) Serum amylase levels in each sample. (e) Serum lipase levels in each sample. (f) Serum TNF‐α levels in each sample. (g) Serum IL‐4 levels in each sample. (h, i) Flow cytometric statistical analysis of infiltrated macrophages and neutrophils in the pancreas between FMT‐SAP and FMT‐HC mice (n = 6). (j) Volcano plot displaying differentially expressed genes in the pancreas between FMT‐SAP and FMT‐HC mice (n = 4). FC fold change. (k) Enriched pathways predicted by differentially expressed genes in the pancreas between FMT‐SAP and FMT‐HC groups using KEGG analysis. (l) Differential genes enriched in cytokine‐cytokine receptor interaction pathway between FMT‐SAP and FMT‐HC mice (n = 4). (m) Workflow strategy diagram for pancreatic immune cells isolation and single‐cell RNA sequencing. (n) A UMAP displaying six distinct cell clusters obtained from FMT‐SAP and FMT‐HC mice. (o) Proportion of macrophage subclusters. (p) Top three marker genes in each macrophage subcluster were shown. *P* values were determined by two‐tailed ordinary one‐way ANOVA with the Tukey post hoc test or Student's *t*‐test. Data was represented as mean ± SEM. *p < 0.05.

To further elucidate the effect of gut microbiota on the SIRS/CARS development, human‐into‐mice fecal microbiota transplantation (FMT) mouse model was built [20, 21]. Mice were pretreated with broad‐spectrum antibiotics (ABX) for one week, followed by subjection to FMT from donors of HC (FMT‐HC), MAP patients (FMT‐MAP) and SAP patients (FMT‐SAP) (Figure 2a). FMT‐SAP exacerbated the pancreatitis toxin and SIRS/CARS development compared with FMT‐MAP and FMT‐HC, regarding to histopathology scores and serum amylase and lipase (Figure 2b‐e). Both FMT‐MAP and FMT‐SAP increased the levels of pro‐inflammatory cytokines (TNF‐α) and anti‐inflammatory cytokines (IL‐4 and IL‐10) compared with FMT‐HC (Figure 2f, g, Figure S2a). FMT‐SAP has a severe representation of pancreatic injury and systemic inflammation than FMT‐MAP. Our previous studies have shown that AP resulted in gut microbiota imbalance and immune disorders [9, 19]. We found increases of macrophages and neutrophils in the pancreas of FMT‐SAP mice compared with FMT‐HC mice (Figure 2h, i, Figure S2b), indicating that manipulation of gut microbiota could become a regulatory scheme for the SIRS/CARS development through immunomodulation.

To explore the underlying mechanisms, the transcriptomic changes in the pancreas between FMT‐HC and FMT‐SAP were compared by bulk RNA sequencing (RNA‐seq). We found 272 upregulated genes and 137 downregulated genes in FMT‐SAP group compared to FMT‐HC group (Figure 2j). KEGG analysis revealed that the cytokine‐cytokine receptor interaction pathway was enriched in the FMT‐SAP group by upregulated genes such as *Pf4*, *Ccl2* and *Cxcl5* (Figure 2k, l). Next, we performed scRNA‐seq in the pancreas of FMT mice (Figure 2m), five main cell types including macrophage, neutrophil, T cell, B cell, dendritic cell were annotated, while macrophage accounted for the largest proportion (Figure 2n, Figure S2d). Macrophages (n = 15 607) affect the progression of SIRS/CARS during AP, which were then subclustered into eight populations (Figure S2c). The largest proportion C0 showed significance between FMT‐SAP group and FMT‐HC group (Figure 2o). The differential genes in C0 cluster were compared between FMT‐SAP group and FMT‐HC group and cytokine‐cytokine receptor interaction pathway, Chemokine signaling pathway and TNF signaling pathway were enriched by upregulated genes using KEGG analysis and GSEA (Figure S2e, f). Next, the marker gene profiles of C0 cluster were visualized and upregulated *Pf4* was found between FMT‐SAP vs FMT‐HC, which was also enriched in the cytokine‐cytokine receptor interaction pathway (Figure 2p, Figure S2e, f). These results were further validated by qPCR assay (Figure S2g). We then annotated C0 cluster as PF4^+^ macrophages. Collectively, our findings suggest that gut microbiota dysbiosis enhances the infiltration of PF4^+^ macrophages in the pancreas that promotes SIRS/CARS development.

### 2.3 PF4^+^ Macrophages Activate the Innate Immune‐Mediated SIRS and the Adaptive Immune‐Mediated CARS in AP

**FIGURE 3:**
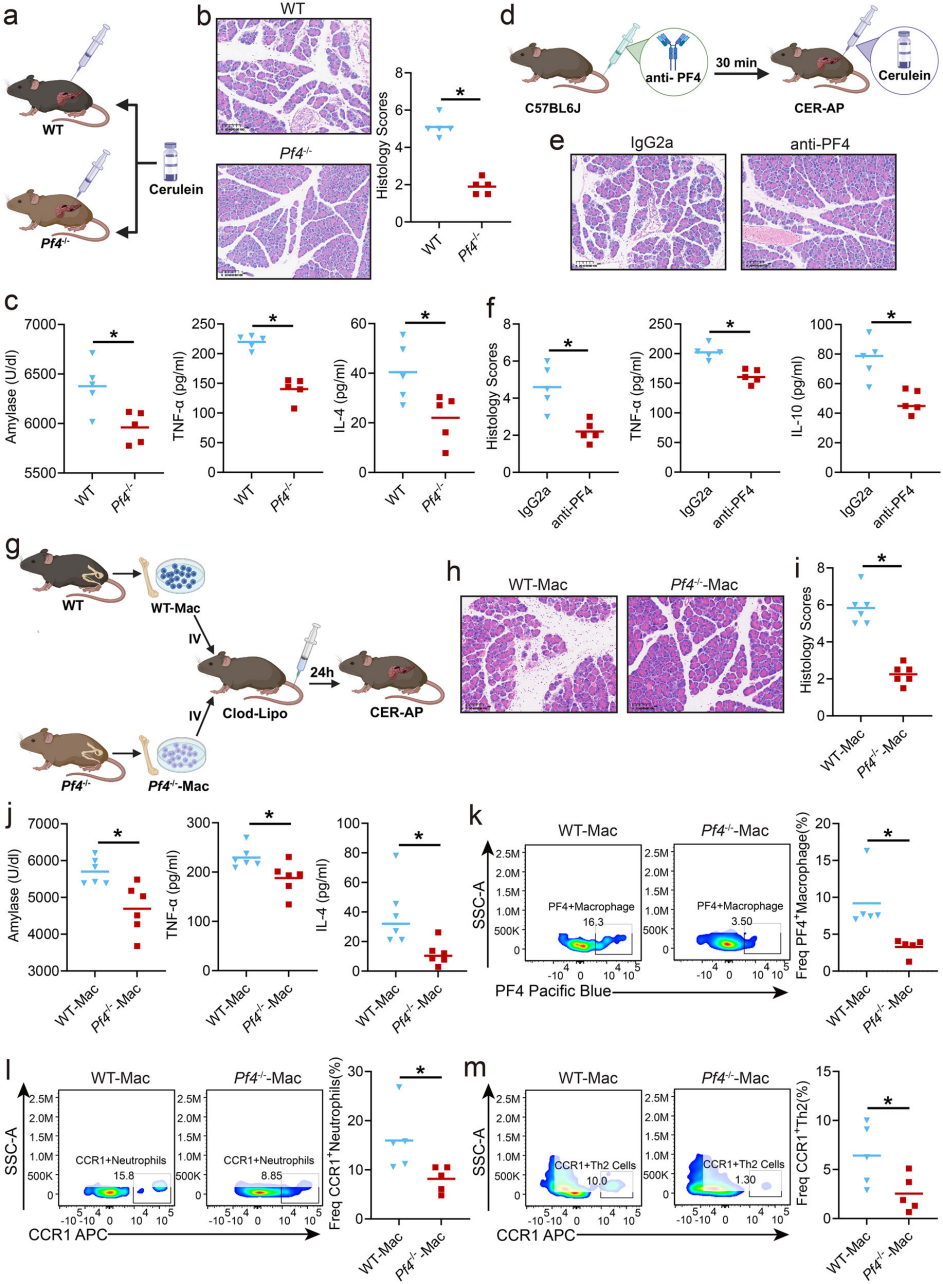
The infiltrated PF4^+^ macrophages determine the SIRS/SARS phenotypes in AP mice. (a) Scheme for *Pf4* ^−/−^ mice treated with caerulein (n = 5). (b) Representative images of Hematoxylin and eosin (H&E) staining and quantification of histology score in the pancreas of *Pf4* ^−/−^ mice (scale bar = 100 µm, n = 5). (c) Serum amylase, TNF‐α and IL‐4 levels in *Pf4* ^−/−^ mice (n = 5). (d) Scheme for AP mice treated with PF4 neutralized antibody (n = 5). (e) Hematoxylin and eosin (H&E) staining in the pancreas of mice treated with PF4‐neutralized antibody (scale bar = 100 µm, n = 5). (f) Histology score of pancreatic tissues in PF4‐neutralized mice (n = 5). Serum TNF‐α and IL‐10 levels in PF4‐neutralized mice (n = 5). (g) Adoptive‐transfer of BMDMs from wildtype mice and *Pf4* ^−/−^ mice into clod‐lipo‐treated mice was performed. (h) Representative images of Hematoxylin and eosin (H&E) staining in the pancreas of BMDMs‐transferred mice (scale bar = 100 µm, n = 6). (i) Histology score of pancreatic tissues in BMDMs‐transferred mice (n = 6). (j) Serum amylase, TNF‐α and IL‐4 levels in each sample (n = 6). (k) Flow cytometric quantification and statistical analysis of infiltrated PF4^+^ macrophages in the pancreas of BMDMs‐transferred mice (n = 5). (l) Flow cytometric quantification and statistical analysis of infiltrated CCR1^+^ neutrophils in the pancreas of BMDMs‐transferred mice (n = 5). (m) Flow cytometric quantification and statistical analysis of infiltrated CCR1^+^ Th2 cells in the pancreas of BMDMs‐transferred mice (n = 5). *P* values were determined by Student's *t*‐test. Data was represented as mean ± SEM. *p < 0.05.

To evaluate whether PF4^+^ macrophages influence SIRS/CARS development during AP, PF4 was either blocked by neutralized mono‐antibody or by genetic perturbation. Genetically PF4 deficiency or PF4 neutralization alleviated pancreatic injury, pro‐inflammatory cytokines (TNF‐α) and anti‐inflammatory cytokines (IL‐4 and IL‐10) (Figure 3a‐f, Figure S3a‐c). Moreover, genetically PF4 deficiency also ameliorated pancreatitis toxin injury in SAP mice (Figure S3d‐h). To further determine whether PF4^+^ macrophages play a specific role in the development of SIRS/CARS, bone marrow‐derived macrophages (BMDMs) from wildtype or *Pf4* ^−/−^ mice were adoptively transferred into wild‐type mice in which macrophages had been indelibly deleted using clodronate‐loaded liposomes (clod‐lipo) (Figure 3g, Figure S3l, m). Transferring PF4^−/−^ macrophages alone suppressed the severity of pancreatic injury and necrosis (Figure 3h, i). Consistently, transferring PF4^−/−^ macrophages to mice with macrophages depletion decreased the levels of serum amylase, pro‐inflammatory cytokines (TNF‐α and IL‐1β) and anti‐inflammatory cytokines (IL‐4 and IL‐10) (Figure 3j, Figure S3n, o). In addition, BMDMs from wild‐type or *Pf4* ^−/−^ mice were adoptively transferred into *Pf4* ^−/−^ mice that had received clod‐lipo (Figure S3p, q). We found that transfer of PF4^+^ macrophages alone increased the severity of pancreatic injury, as well as the levels of serum amylase, lipase and IL‐1β (Figure S3r–v). These indicate that PF4^+^ macrophages infiltrating the inflamed pancreas contributes to SIRS/CARS progression.

To investigate the mechanism of PF4^+^ macrophages modulating systemic inflammation, upregulated genes in C0 cluster were identified, which resulted in neutrophils recruitment and T cells activation, indicating that PF4^+^ macrophages may interact with neutrophils and T cells in SIRS/CARS development (Figure S4a). We then found that the proportion of neutrophils in the FMT‐SAP group upregulated compared with the FMT‐HC group (Figure S4b, c). The proportion of CD4^+^ T cells and Th2 cells were upregulated in the FMT‐SAP group compared to FMT‐HC group (Figure S4d, e), while no differences in CD8^+^ T cells, Th1 cells, Th17 cells, and Treg cells. PF4 can communicate with its receptors including CCR1, LRP1, and CXCR3 that trigger the inflammatory signals [22, 23, 24, 25]. To further investigate whether PF4^+^ macrophages can recruit neutrophils and Th2 cells through ligand‐receptor interaction, the levels of those receptors in the neutrophils and Th2 cells were compared between FMT‐HC group and FMT‐SAP group. We found that the levels of *Ccr1* in the neutrophils and Th2 cells were upregulated in the FMT‐SAP group, while there were no differences in the expressions of *Lrp1* and *Cxcr3* (Figure S4f, g). Flow cytometry indicated that the proportion of infiltrating PF4^+^ macrophages, CCR1^+^ Th2 cells and CCR1^+^ neutrophils were downregulated in AP mice that had received PF4^−^ macrophages or were genetically PF4 deficient (Figure 3k‐m, Figure S3i‐k). To further investigate the role of CCR1 in the recruitment of Th2 cells and neutrophils by PF4^+^ macrophages, AP mice were treated with CCR1 inhibitor BX471, and pancreatic injury and immune activation were subsequently assessed (Figure S4h). BX471 administration ameliorated pancreatic injury and downregulated serum levels of amylase, lipase and IL‐1β (Figure S4i‐m). In addition, BX471 decreased the proportion of CCR1^+^ neutrophils and CCR1^+^ Th2 cells in the inflamed pancreas, while the infiltration of pancreatic PF4^+^ macrophages remained unchanged (Figure S4n‐p). These data suggest that PF4^+^ macrophages recruit neutrophils and Th2 cells to the inflamed pancreas through the PF4‐CCR1 axis, which further promote the development of SIRS/CARS.

### 2.4 *B.thetaiotaomicron* Alleviates SIRS/CARS Phenotype by Inhibiting the PF4^+^ Macrophages ‐Mediated Immune Activation

**FIGURE 4:**
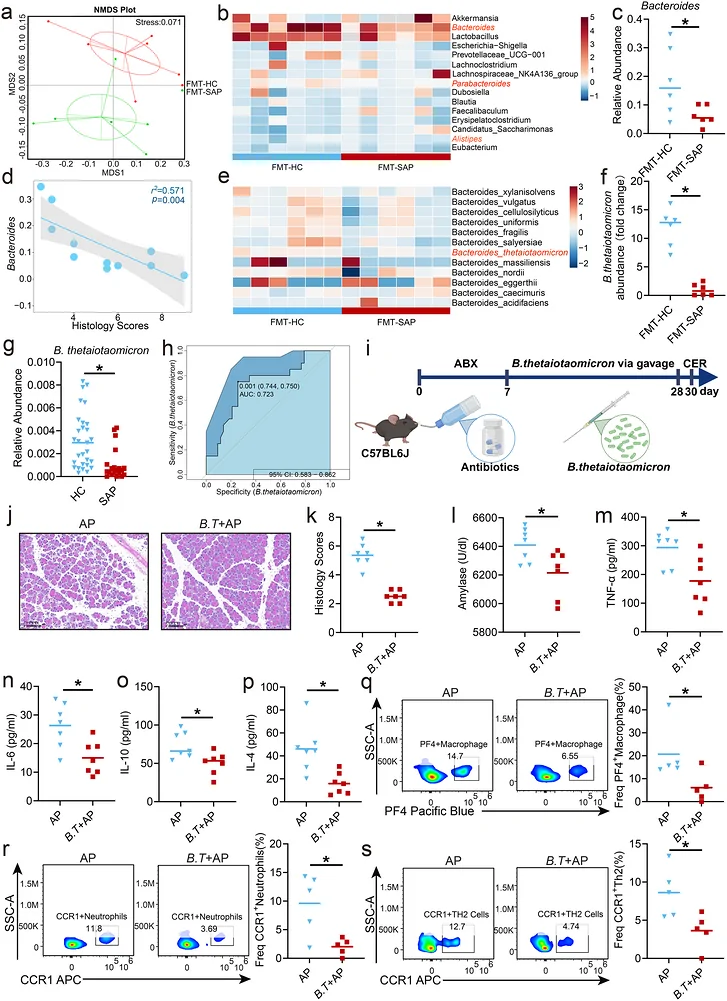
*B.thetaiotaomicron* suppresses PF4^+^ macrophages‐mediated SIRS/CARS phenotype in AP mice. (a) Nonmetric multidimensional scaling (NMDS) plot clustering differential microbial distributions between FMT‐SAP mice and FMT‐HC mice (n = 6). (b) Relative abundance of the top 15 most abundant genera in each sample. (c) Relative abundance of the genera *Bacteroides* in each sample. (d) Correlation analysis between the abundance of *Bacteroides* and histology score (95% confidence interval: −0.9275 to −0.3214). (e) Relative abundance of *Bacteroides* species in each sample. (f) The relative expression of *B.thetaiotaomicron* in the FMT‐treated mice using qPCR (n = 6). (g) Relative abundance of *B.thetaiotaomicron* in the clinical samples (HC, n = 29; SAP, n = 20). (h) The ROC curve discriminating SAP vs HC volunteers using the abundance of *B.thetaiotaomicron*. (i) Schematic representation of *B.thetaiotaomicron* supplementation experiment. AP mice were pre‐treated with antibiotics and then randomly divided into AP group and *B.thetaiotaomicron* (*B.T*)+ AP group. (j, k) Representative images of Hematoxylin and eosin (H&E) staining and quantification of histology score in the pancreas of *B.thetaiotaomicron*‐treated mice (scale bar = 100 µm, n = 7). (l‐p) Serum amylase, TNF‐α, IL‐6, IL‐10 and IL‐4 levels in each sample (n = 7). (q) Flow cytometric quantification and statistical analysis of infiltrated PF4^+^ macrophages in the pancreas between *B.thetaiotaomicron*‐treated mice and PBS‐treated mice (n = 5). (r) Flow cytometric quantification and statistical analysis of infiltrated CCR1^+^ neutrophils in the pancreas between *B.thetaiotaomicron*‐treated mice and PBS‐treated mice (n = 5). (s) Flow cytometric quantification and statistical analysis of infiltrated CCR1^+^ Th2 cells in the pancreas between *B.thetaiotaomicron*‐treated mice and PBS‐treated mice (n = 5). *P* values were determined by two‐tailed Student's *t*‐test. Data was represented as mean ± SEM. *p < 0.05.

To identify which bacterial species is responsible for PF4^+^ macrophages‐mediated SIRS/CARS, the gut microbiota of AP patients and recipient mice were cross‐compared. Sequencing gut microbiota after FMT revealed significant β‐diversity differences between the FMT‐HC group and FMT‐SAP group, with a reduced abundance of *Bacteroides* in the FMT‐SAP group (Figure 4a‐c). We found a negative correlation between the abundance of *Bacteroides* and pancreatic injury in the FMT mouse models (Figure 4d). At species level, *Bacteroides_thetaiotaomicron* (*B.thetaiotaomicron*) was the only one of most significant taxa that were decreased in FMT‐SAP mice (Figure 4e, f). In addition, the abundance of *B.thetaiotaomicron* was downregulated in SAP patients and exhibited higher sensitivity and specificity in indicating AP severity (Figure 4g, h). To investigate whether lacking *B.thetaiotaomicron* can exacerbate SIRS/CARS progression, *B.thetaiotaomicron* was then supplemented in AP mice (Figure 4i, Figure S5a), we found that *B.thetaiotaomicron* reduced pancreatic injury (Figure 4j, k), downregulated the levels of serum amylase, proinflammatory cytokines (TNF‐α and IL‐6), and anti‐inflammatory cytokines (IL‐10 and IL‐4) (Figure 4l‐p). In addition, *B.thetaiotaomicron* suppressed the pancreatic infiltration of PF4^+^ macrophages, CCR1^+^ Th2 cells and CCR1^+^ neutrophils, accompanied by a marked reduction in pancreatic PF4 expression (Figure 4q‐s, Figure S5b‐d). Next, SAP models were induced by employing duct ligation in combination with caerulein injection or sodium taurocholate treatment, we found that *B.thetaiotaomicron* supplementation reduced pancreatic injury and decreased PF4^+^ mcrophages, CCR1^+^ neutrophils and CCR1^+^ Th2 cells in both SAP models (Figure S5e‐q). These suggest that AP results in the downregulation of *B.thetaiotaomicron* that enhances PF4^+^ macrophages‐mediated immune dysfunction and exacerbates SIRS/CARS.

### 2.5 *B.thetaiotaomicron*‐Derived 1MNA Reduces PF4^+^ Macrophages‐Mediated Immune Activation in AP

**FIGURE 5:**
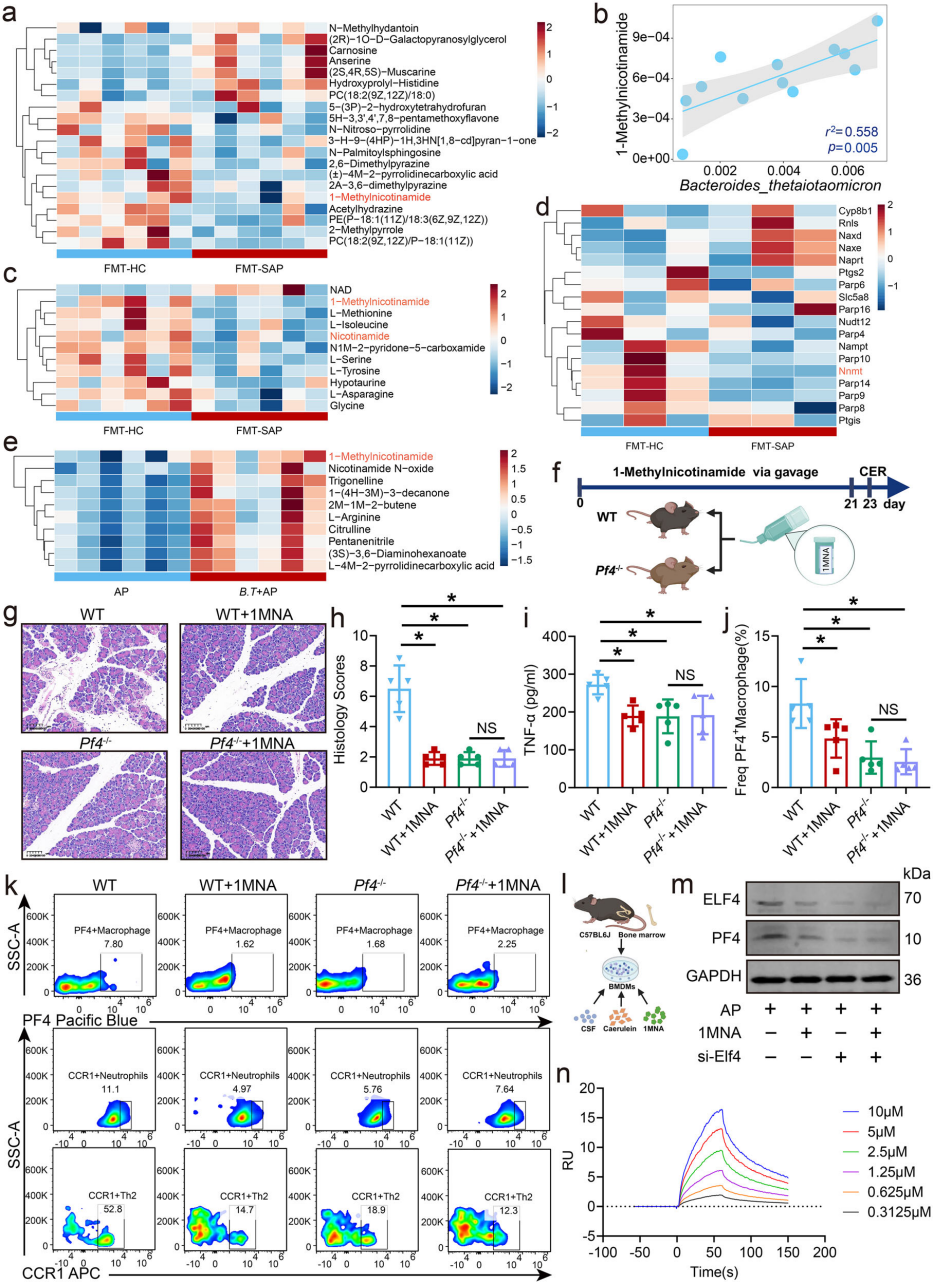
*B.thetaiotaomicron* upregulates intestinal 1MNA and suppresses SIRS/CARS phenotype by targeting PF4^+^ macrophages. (a) Heatmap plotting different serum metabolites between FMT‐SAP and FMT‐HC mice (n = 6). (b) Correlation analysis between the abundance of *B.thetaiotaomicron* and serum 1MNA levels using Spearman's correlation analysis. (c) Heatmap plotting different gut metabolites between FMT‐SAP and FMT‐HC mice (n = 6). (d) Heatmap plotting different genes in the colon between FMT‐SAP and FMT‐HC mice (n = 3). (e) Heatmap plotting different serum metabolites between *B.T*+ AP group and AP group (n = 6). (f) Schematic representation of 1MNA supplementation in wildtype mice and *Pf4* ^−/−^ mice. (g) Representative images of Hematoxylin and eosin (H&E) staining in the pancreas (scale bar = 100 µm, n = 5). (h) Histology score of pancreatic tissues (n = 5). (i) Serum TNF‐α levels in each sample (n = 5). (j) Flow cytometric statistical analysis indicating the percentage of PF4^+^ macrophages in the pancreas (n = 5). (k) Flow cytometric quantification of infiltrated PF4^+^ macrophages, CCR1^+^ neutrophils and CCR1^+^ Th2 cells infiltration in each sample (n = 5). (l) Schematic representation of 1MNA supplementation in the BMDMs from mice. (m) Representative western blot images of ELF4 and PF4 proteins in macrophages treated with si‐ELF4 and 1MNA (n = 5), original blots were found in Figure S12a. (n) The SPR of 1MNA and ELF4 protein. *P* values were determined by two‐tailed ordinary one‐way ANOVA with the Tukey post hoc test or Student's *t*‐test. Data was represented as mean ± SEM. *p < 0.05.

Metabolomics was the crucial portion in bridging the gut microbiota and immune homeostasis [11, 26], serum metabolites from FMT mouse models were then profiled (Figure 5a). We found that 1‐methylnicotinamide (1MNA) was increased in the FMT‐HC group compared to FMT‐SAP group (Figure 5a). The correlation analysis between gut microbiota and host metabolites was performed and the upregulation of 1MNA was positively correlated with increased abundance of *Bacteroides* and *B.thetaiotaomicron* (Figure 5b, Figure S6a). 1MNA is produced by nicotinamide N‐methyltransferase (NNMT) transferring a methyl group from S‐adenosyl‐l‐methionine (SAM) to nicotinamide (NAM) [27] (Figure S6b), we found that *B.thetaiotaomicron* harbors sequences of *nada* and *metk*, encoding the 1MNA precursor NAM and SAM (Figure S6c). Since *B.thetaiotaomicron* was enriched in the colon [28], untargeted metabolomics of colon tissues showed that FMT‐HC increased the levels of NAM and 1MNA compared to FMT‐SAP, while SAM exhibited an increasing trend (Figure 5c, Figure S6d, e). RNA‐seq analysis revealed upregulated NNMT expression in the colon tissues of FMT‐HC mice, suggesting that *B.thetaiotaomicron* enhances intestinal NNMT expression and consequently increases 1MNA levels (Figure 5d, Figure S6f). Consistently, *B.thetaiotaomicron* administration upregulated the colonic NNMT expression and serum 1MNA levels in AP mice (Figure 5e, Figure S6g). These findings suggest that *B.thetaiotaomicron* upregulates the host's 1MNA content by increasing the production of its precursor metabolites and enhancing the level of key enzyme.

To investigate whether *B.thetaiotaomicron*‐derived 1MNA alleviates SIRS/CARS through PF4^+^ macrophages, AP model was built by 1MNA‐pre‐treated *Pf4* ^−/−^ mice, pancreatic injury and infiltrated immune cells were detected (Figure 5f). Supplementation with 1MNA or PF4 deficiency ameliorated pancreatic injury and decreased serum levels of amylase, lipase, proinflammatory cytokines, and anti‐inflammatory cytokines in AP and SAP models (Figure 5g‐i, Figures S6h‐l and S7a‐c, h‐j), while the protective effect of 1MNA was abolished in *Pf4* ^−/−^ mice (Figure 5g‐i, Figure S6h‐l). Flow cytometry showed decreased proportions of PF4^+^ mcrophages, CCR1^+^ neutrophils and CCR1^+^ Th2 cells after 1MNA administration or in the *Pf4* ^−/−^ mice (Figure 5j, k, Figures S6m, n, and S7d‐g, k‐n). We also found that administration of 1MNA cannot reverse AP‐induced immune activation in mice without PF4^+^ macrophages (Figure 5j, k, Figure S6m, n). To explore the mechanism by which 1MNA restrains the activation of PF4^+^ macrophages, we screened the known transcription factors of PF4 in macrophages using scRNA‐seq analysis and found that *Pbx2*, *Fli1*, *Runx1* and *Elf4* were upregulated after FMT‐SAP compared to FMT‐HC (Figure S8a) [29, 30]. Western blotting analysis indicated that the pancreatic expressions of PF4 and ELF4 were decreased after 1MNA administration (Figure S8b‐d). To investigate the regulatory role of 1MNA on ELF4 transcriptional activity, luciferase reporter assays using ELF4 and PF4 promoters were performed, showing that ELF4 drove PF4 transcription (Figure S8g). Molecular dynamics simulation suggested that 1MNA could coordinate within the ELF4 cavity, and surface‐plasmon resonance analysis confirmed a dose‐dependent direct interaction between 1MNA and ELF4, indicating a high binding affinity (Figure S8h, i, Figure 5n). To explore whether the effect of 1MNA is specifically achieved through ELF4 in macrophages, BMDMs were transfected with ELF4 siRNA to knockdown ELF4 expression and then treated with 1MNA. Western blotting and qPCR analysis indicated that 1MNA supplementation decreased ELF4 and PF4 expression in macrophages, whereas this effect was lost in the ELF4‐knockdown cells (Figure 5m, Figure S8j‐m). In addition, we established a myeloid‐specific ELF4 knockout model using intraperitoneal injection of adeno‐associated virus type 8 (AAV)‐MacElf4 [31]. AAV‐*MacElf4* injection specifically depleted the infiltration of ELF4^+^ macrophages in the inflamed pancreas, while 1MNA also reduced the infiltration of ELF4^+^ macrophages (Figure S9a). Both 1MNA supplementation and AAV*‐MacElf4* injection ameliorated pancreatic injury and decreased serum levels of amylase, TNF‐α and IL‐4, while the protective effect of 1MNA was abolished by AAV*‐MacElf4* injection (Figure S9b‐f). Flow cytometry showed decreased proportions of PF4^+^ macrophages, CCR1^+^ Th2 cells and CCR1^+^ neutrophils after 1MNA administration or AAV*‐MacElf4* treatment (Figure S9g‐j). We also found that 1MNA cannot reverse SAP‐induced immune activation in mice lacking of ELF4^+^ macrophages (Figure S9g‐j). Taken together, these findings suggest that *B.thetaiotaomicron*‐derived 1MNA directly inhibits the transcription of PF4 by targeting ELF4 in macrophages, thereby alleviating the SIRS/CARS phenotype.

### 2.6 *B.thetaiotaomicron*‐Derived 1MNA Suppresses PF4^+^ Macrophages‐Mediated SIRS/CARS Phenotype in AP Patients

**FIGURE 6:**
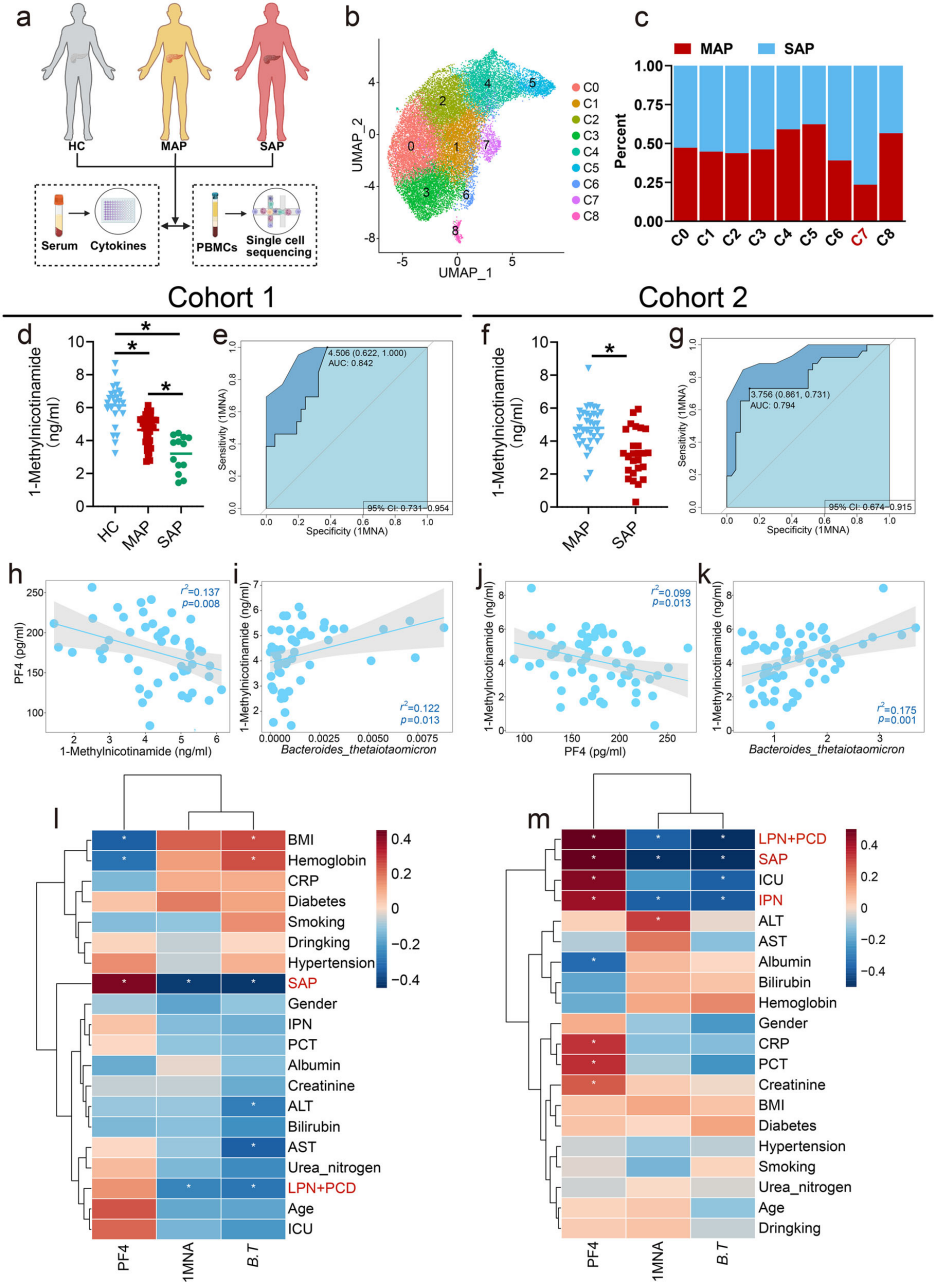
*B.thetaiotaomicron*‐derived 1MNA is associated with PF4^+^ macrophages‐mediated immune response in AP patients. Human feces and blood samples were collected from two independent cohorts, including cohort 1 (year, 2020–2022; HC, n = 29; MAP, n = 43; SAP, n = 20) and cohort 2 (year, 2023–2026; MAP, n = 36; SAP, n = 26). (a) Schematic diagram of hPBMCs collection for scRNA‐seq and serum collection for ELISA assay. (b) A UMAP displaying distinct clusters of monocytes in hPBMCs. (c) Proportion of monocytes subclusters. (d) Serum 1MNA levels of each human sample in cohort 1. (e) The ROC curve distinguishing SAP vs MAP patients in cohort 1 using 1MNA level. (f) Serum 1MNA levels of each human sample in cohort 2. (g) The ROC curve distinguishing SAP vs MAP patients in cohort 2 using 1MNA level. (h) Correlation analysis between the serum PF4 levels and 1MNA levels in AP patients from cohort 1 (95% confidence interval: ‐0.5881 to ‐0.1026). (i) Correlation analysis between the abundance of *B.thetaiotaomicron* and the serum 1MNA levels in AP patients from cohort 1 (95% confidence interval: 0.07910 to 0.5724). (j) Correlation analysis between the serum PF4 levels and 1MNA levels in AP patients from cohort 2 (95% confidence interval: ‐0.5239 to ‐0.07127). (k) Correlation analysis between the abundance of *B.thetaiotaomicron* and the serum 1MNA levels in AP patients from cohort 2 (95% confidence interval: 0.1882 to 0.6049). (l) Correlation analysis between fecal *B.thetaiotaomicron* abundance, serum 1MNA and PF4 levels and clinical index in cohort 1. (m) Correlation analysis between fecal *B.thetaiotaomicron* abundance, serum 1MNA and PF4 levels and clinical index in cohort 2. *P* values were determined by two‐tailed ordinary one‐way ANOVA with the Tukey post hoc test or Student's *t*‐test. All correlation analysis was tested with Spearman's correlation. Data was represented as mean ± SEM. *p < 0.05.

Circulating monocytes can migrate to the pancreas and differentiate into macrophages when AP occurs [5, 32], we then explored whether the PF4^+^ monocytes could be used as a biomarker for predicting the severity of disease in AP patients (Figure 6a). Using our scRNA‐seq dataset, the monocytes were re‐clustered and nine subclusters were annotated, including C0 (*S100A12*), C1 (*NEAT1*), C2 (*S100A10*), C3 (*FAM101B*), C4 (*HLA‐DPA1*), C5 (*FCGR3A*), C6 (*MALAT1*), C7 (*PF4*) and C8 (*CCL5*) (Figure 6b, Figure S10a). We found that PF4^+^ monocytes were upregulated in SAP patients compared to MAP patients (Figure 6c, Figure S10a). GO analysis of PF4^+^ monocytes revealed that, compared to MAP patients, the upregulated genes in SAP patients were enriched in immune system process and inflammatory response (Figure S10b). To verify the role of “gut microbiota‐metabolites‐immune” axis in AP patients, we collected human fecal and blood samples from two independent cohorts. The serum PF4 levels was upregulated in SAP patients and exhibited higher sensitivity and specificity in indicating AP severity (Figure S10c‐h). Similarly, the downregulated serum 1MNA levels and *B.thetaiotaomicron* abundance in SAP patients suggested their high sensitivity and specificity in reflecting AP severity (Figure 6d‐g, Figure S10i, j, Figure 4g, h). We found that serum 1MNA levels were positively correlated with *B.thetaiotaomicron* abundance and negatively correlated with serum PF4 levels (Figure 6h‐k). There was an inverse correlation between serum PF4 levels with the abundance of *B.thetaiotaomicron* (Figure S10k‐m). Next, we explored the clinical relevance of fecal *B.thetaiotaomicron* abundance, serum 1MNA and PF4 levels in AP patients and found that both *B.thetaiotaomicron* abundance and 1MNA levels were negatively correlated with the incidence of SAP and infected pancreatic necrosis, while serum PF4 levels were positively correlated with these clinical outcomes (Figure 6l, m). Flow cytometry revealed that PF4^+^ Monocytes and Treg cells were upregulated in SAP patients compared to MAP patients (Figure S10g, h, n). The proportion of PF4^+^ Monocytes and Treg cells was positively associated with the AP severity (Figure S10o). These suggest that *B.thetaiotaomicron*‐derived 1MNA inhibits the PF4 expression in macrophages, which alleviates SIRS/CARS development in AP patients.

## 3 Discussion

The systemic phenomenon of AP deterioration can be subdivided into the proinflammatory hyperinflammation phase (SIRS) and the hyperinflammatory response phase (CARS) [6, 33, 34]. SIRS occurs in the early phase which is associated with local complications and organ failures, is the leading cause of high mortality [6]. CARS is a consequence of SIRS, which manifests as immune deficiency or suppression, leads to higher susceptibility to pancreatic and peripancreatic tissue infections [6, 7]. The imbalance of gut microbiota was caused by AP, while the role of gut microbiota dysbiosis on the development of SIRS/CARS remains unclear. Using human‐into‐mice FMT models, we found that colonization of gut microbiota obtained from SAP patients resulted in downregulation of *Bacteroides* which is recognized as main cause of exacerbating pancreatitis toxin and SIRS/CARS. *Bacteroides* species such as *Bacteroides_uniform* and *Bacteroides_stercoris* regulate pancreatic injury and pancreatic islet dysfunction [9, 35]. Here, we identified *B.thetaiotaomicron* as a predominant colonizing species, which was downregulated in AP patients, as well as in the recipient mice after FMT‐SAP from a small sample size FMT study. *B.thetaiotaomicron* can activate mucosa and host immunity though microbial‐host interaction or metabolites which plays beneficial roles in cancer, inflammatory and autoimmune diseases [36, 37, 38, 39]. Supplementation of *B.thetaiotaomicron* ameliorated pancreatitis injury and reversed SIRS/CARS phenotype, suggesting its potential for SIRS/CARS prevention.

The “gut microbiota‐immune response” axis influences AP deterioration and associated SIRS [8, 9, 19, 34, 40]. Gut microbiota dysbiosis promote macrophage polarization and neutrophil extracellular traps formation that trigger SIRS [8, 9, 40]. AP and sepsis induced immunosuppression impairs the clearance of primary injury and predisposes patients to lethal secondary infections that promote the development of SIRS/CARS [41, 42, 43, 44, 45]. Macrophages can migrate to the pancreas and exhibit different roles depending on their subtypes [46, 47], we identified PF4^+^ macrophages as key population accelerating disease exacerbation through the recruitment of neutrophils‐associated SIRS and triggered Th2 cells‐mediated CARS. PF4 is a platelet specific CXC chemokine that forms tetramers with a compact globular structure and a strong equatorial positive charge, enabling high‐affinity binding to negatively charged molecules such as endothelial proteoglycans and infectious agents [48]. Through these interactions, PF4 regulates megakaryocyte colony formation and platelet aggregation [48, 49]. Previous studies have shown that PF4 interacts with chemokine receptors to recruit and activate neutrophils, Th cells, CD8^+^ T cells, contributing to the pathogenesis of ulcerative colitis and cancer [25, 50, 51]. In addition, PF4^+^ macrophages have been implicated in cancer, ocular disease and renal fibrosis [52, 53, 54]. Our study revealed that CCR1 mediates the recruitment of neutrophils and Th2 cells to the pancreas downstream of PF4^+^ macrophages, which contribute to the AP progression. Importantly, FMT and *B.thetaiotaomicron* administration suppressed the cross‐talks between innate and adaptive immune response by targeting PF4^+^ macrophages, suggesting that targeting commensal bacteria can evoke immune system mediated protection and maintain homeostasis, which in turn restrains the development of SIRS/CARS.

Gut microbiota modulates the host immune response‐mediated diseases progression through metabolites, either directly produced microbial metabolism or indirectly generated host's metabolism within gut epithelial cells [8, 9, 19, 55]. Our findings suggest that colonization of *B.thetaiotaomicron* enhanced the local and systemic levels of 1MNA. 1MNA is formed by NNMT transferring a methyl group from SAM to NAM [27]. *B.thetaiotaomicron* contains a gene operon‐encoding methylmalonyl‐CoA mutase (MCM) that increases the biosynthesis of propionate [36]. Similarly, the genome of *B.thetaiotaomicron* contains ORF01166 encoding *nada* and *metk*, which are key enzymes for the biosynthesis of 1MNA precursor NAM and SAM. We demonstrated that *B.thetaiotaomicron* upregulated the levels of 1MNA in the serum by enhancing the biosynthesis of 1MNA precursors and 1MNA synthase activity in the colonic epithelial cells. 1MNA plays a protective role in various inflammatory diseases [56, 57, 58], in our study, 1MNA administration ameliorated both pro‐inflammatory cytokines‐associated SIRS and anti‐inflammatory cytokines‐mediated CARS, suggesting that *B.thetaiotaomicron*‐derived 1MNA alleviates immune dysregulation‐driven SIRS/CARS.

1MNA inhibits the activation of NLRP3 inflammasome in macrophages which enhances intestinal barrier function [56, 59]. 1MNA upregulates the abundance of *Blautia* in the gut which balances the ratio of Th17/Treg cells and alleviates diabetes [60]. Our data illustrates that 1MNA inhibits the infiltration of PF4^+^ macrophages into the damaged pancreas, ameliorating innate immune system‐mediated SIRS through the regulation of neutrophil recruitment. Meanwhile, 1MNA‐inhibited PF4^+^ macrophages acts as a regulator of Th2 cell responses, alleviating the adaptive immune system‐mediated CARS. Previous study has shown that 1MNA enhances the transcriptional binding of Sp1 to the TNF‐α promoter in T cells, which drive tumorigenesis [61]. The transcription factors of PF4 bind to the PF4 promoter regions, activating its transcription. To date, eight such transcription factors have been identified in the host [29, 30, 62, 63, 64, 65]. We recognized ELF4 as a key transcription factor of PF4 in macrophages which explains the causal relationship between 1MNA and PF4^+^ macrophages induced‐immune disorders during AP. Our data raises the novel aspects of 1MNA suppressing the infiltration of PF4^+^ macrophages in the pancreas that exacerbated SIRS/CARS.

Our study expands the view of gut microbiota‐derived epithelium metabolism influencing innate immune response‐mediated pro‐inflammatory status and adaptive immune response‐mediated anti‐inflammatory response, suggesting that *B.thetaiotaomicron*‐based probiotic supplements and 1MNA‐associated dietary intervention can be utilized as promising therapeutic approaches for SIRS/CARS.

## 4 Methods

### 4.1 Human Subjects and Sample Collection

The human study was approved by the Ethics Committee of the First Affiliated Hospital of Harbin Medical University (2020XS32‐02), and all participants signed the informed consent. We collected human fecal and blood samples from two independent cohorts, including cohort 1 (year, 2020–2022; healthy volunteers, n = 29; mild acute pancreatitis, n = 43; severe acute pancreatitis, n = 20) and cohort 2 (year, 2023–2026; mild acute pancreatitis, n = 36; severe acute pancreatitis, n = 26). No differences of baseline information (including age, sex and so on) were found within two independent cohorts (Tables S1 and S2).

Acute pancreatitis was diagnosed and classified by the 2012 revised Atlanta criteria [66]. The exclusion criteria were: patients <18 years; pregnancy; patients with chronic pancreatitis; inflammatory bowel disease; cancer; irritable bowel syndrome; gastroenteritis; diseases judged and taking antibiotics, pro‐biotics, pre‐biotics, proton‐pump inhibitors, laxatives during the last 2 months. Human serum, feces and PBMCs samples were collected on the first day of hospital admission. The blood samples were centrifuged to separate the supernatant and obtain the serum. The blood samples were mixed with Percoll and centrifuged to separate cell layers, thereby obtaining PBMCs. Feces samples were collected and immediately stored at −80°C until further analysis. The clinical characteristics of participants was shown in Tables S1 and S2.

### 4.2 Animals and Experimental Model

Six‐week to eight‐week‐old male C57BL/6 mice were purchased from Liaoning Changsheng Biotechnology Co. Ltd. (Liaoning, China). *Pf4* ^−/−^ mice was purchased from GemPharmatech Co. Ltd. (Jiangsu, China). Age‐ and sex‐matched wild‐type C57BL/6J mice were used as controls. All animals were housed in specific pathogen‐free environment with standard conditions (including a constant temperature 22 ± 2°C, 12 h light/dark cycle and 45 ± 5% humidity) for at least 1 week before any study procedures were performed. The animals had ad libitum access to sterile water and standard chow diet. All animal experiments were approved by the Institutional Animal Ethics Committee of The First Affiliated Hospital of Harbin Medical University (Heilongjiang, China). The Institutional Animal Care and Use Committee (IACUC) number is 2021143.

AP was performed by administering 12 hourly intra‐peritoneal injection of caerulein (Sigma‐Aldrich, St. Louis, MO, USA; 50 µg/kg/bodyweight), whereas the control mice received phosphate‐buffered saline (PBS) [19]. CL‐SAP model was induced by partial pancreatic duct ligation, followed by an additional dose of caerulein (50 µg/kg/bodyweight) injection two days later [6]. Tau‐SAP model was infused with 50 ul of 2.5% sodium taurocholate into the bile‐pancreatic duct and sacrificed 24 h later [67]. The animals were euthanized by CO_2_ suffocation at 12 h following the first injection. Blood, pancreas, cecum tissue and feces were collected for further analysis. The collected serum and feces were stored at −80°C. Pancreas were removed and either fixed in 4.5% formaldehyde, embedded in paraffin for histological analysis or were shock frozen in liquid nitrogen and stored at −80°C. pancreas and cecum were collected for flow cytometry and metabolomics analysis, and were homogenized immediately for RNA sequencing.

### 4.3 Fecal Microbiota Transplantation (FMT)

Fresh fecal samples were collected from three healthy controls, three MAP patients, and three SAP patients. The FMT program involves three mice corresponding to one donor. Their clinical characteristics were matched and presented in Table S3. The fecal microbiota suspensions were resuspended in pre‐chilled PBS at a concentration of 100 mg/mL, and then filtered through a sterile 70 µm pore mesh. The fecal microbiota suspensions from each donor were transplanted into three recipient mice, which were housed in the same cage. To deplete the gut microbiota, streptomycin (Sigma‐Aldrich, St. Louis, MO, USA; 5 mg/mL) and clindamycin (Sigma‐Aldrich, St. Louis, MO, USA; 0.1 mg/mL) were dissolved in drinking water and provided to the recipient mice for seven days [68]. Then, 200 µl of microbiota supernatant was orally administered to a recipient mouse (3 times a week for 3 consecutive weeks), while the control mice were gavage with sterile PBS.

### 4.4 16S Ribosomal RNA Sequencing and Analysis

Total bacterial DNA of feces was extracted using the Power Soil DNA Isolation Kit (MO BIO Laboratories). The V3‐V4 length sequences was amplified using the common primers (Forward primer, 5’‐ACTCC‐TACGGGAGGCAGCA‐3’; reverse primer, 5’‐GGACTACHVGGGTWTC‐TAAT‐3’) combined with adapter sequences. The PCR products were mixed with an equal volume of 1×loading buffer (contained SYBR green) and electrophoresed on 2% agarose gel. PCR products were mixed in equal ratios, and Qiagen Gel Extraction Kit (Qiagen, Germany) was used to purify the mixture PCR products. The sequencing libraries were generated using NEBNextUltra IIDNA Library Prep Kit (Cat No. E7645) according to the manufacturer's instruction manual. Finally, the library was sequenced on Illumina NovaSeq platform.

The raw FASTQ files was obtained as described previously. Denoising was conducted using the DADA2 or deblur module in the QIIME2 software (Version QIIME2‐ 202006) to generate amplicon sequence variants (ASVs). Sequences were filtered for the minimum abundance 5. The taxonomic annotation for bacteria at the species resolution was generated according to SILVA database and QIIME2 software. The absolute abundance of gut microbiota was normalized with a standard of sequence number corresponding to the sample with the least sequences. The alpha diversity and beta diversity were calculated using the above data.

### 4.5 Untargeted Metabolomics Analysis

For the LC‐MS system analysis of intestinal tissue, approximately 20 mg tissue were homogenized in 100 mL sterile water and then centrifuged at 3500 g for 10 min. Mice blood samples were centrifuged at 15 000 g for 20 min. The supernatant of intestine and blood was injected into the LC‐MS system analysis. The raw data files generated by LC‐MS were processed using Compound Discoverer to perform peak alignment, peak picking and quantification. Next, the peak value was normalized to the total spectral intensity. The peaks were matched with KEGG database, HMDB database and LIPIDMaps database, which contributed to obtain accurate qualitative results. The statistical software R, Python and CentOS were used for statistical analysis.

### 4.6 Single‐Cell RNA Sequencing

Fresh pancreatic tissue from FMT‐treated mice was digested in 2 mL GEXSCOPE Tissue Dissociation Solution (Singleron Biotechnologies) for 15 min at 37°C. Subsequently, the digestion was terminated by adding washing buffer that contains fetal bovine serum. Single cell suspensions were obtained after going through 40‐micron sterile strainer (Corning) and centrifuged at 300 g for 5 min. The blood samples were mixed with Percoll and used density gradient centrifugation at 800 g for 30 min, thereby obtaining PBMCs single cell suspensions. For all the samples, the cell numbers and the cell viability were collected by CountStar. Single‐cell suspensions were loaded to microfluidic chip (GEXSCOPE Single Cell RNA‐seq Kit, Singleron Biotechnologies) [69]. The scRNA‐seq libraries were sequenced on the NovaSeq 6000 System (Illumina) with 150 bp paired‐end sequencing of reads. Dead cells with a mitochondrial percentage greater than 25% were excluded.

### 4.7 RNA Sequencing Analysis

Fresh pancreatic samples and colonic samples were performed for RNA sequencing. All samples were cleaned up using RNeasy kit (Qiagen China (Shanghai) Co Ltd, China) and performed for quality by the RNA Nano 6000 Assay Kit of the Bioanalyzer 2100 system (Agilent Technologies, CA, USA). All samples were performed on the Illumina NovaSeq 6000 (Illumina, San Diego, CA, USA). DeSeq2 was used to analysis RNA sequencing. Reads were matched to the mice genome based on TopHat v2.0.11 (http://tophat.cbcb). Read counts were merged into a count file for further differential expression analysis.

### 4.8 *B.thetaitaomicron* Culture and Supplement

Human‐derived *B.thetaiotaomicron* VPI‐5482 was purchased from American type culture collection (ATCC, USA). *B.thetaiotaomicron* was cultured in the brain heart infusion (BHI) broth (Hopebio, Qingdao, China) at 37°C under anaerobic condition [28]. Antibiotics pre‐treated mice were gavage with *B.thetaiotaomicron* solution (1 × 10^9^ CFU/200 µL per mice) or equivalent sterile PBS (control group) every two days for three consecutive weeks [70]. One day after the last dose of oral gavage, AP models were then built.

### 4.9 1MNA Measurement and Supplement

1MNA (CAS: 1005‐24‐9, MCE, Shanghai, China) was dissolved in PBS. Mice were gavage with 1MNA (100 mg/kg body weight) or an equivalent amount of sterile PBS (control group) every day for three consecutive weeks [58]. The serum 1MNA was measured using enzyme‐linked immunosorbent assay kit (Meimian, Jiangsu, China) according to the manufacturer's instruction.

### 4.10 AAV8 Vector Transduction

The AAV8 vector (Genomeditech, Shanghai, China) was injected into mice via intraperitoneal injection to evaluate the therapeutic efficacy of the target gene mediated by the AAV8 vector in AP mice. Specifically, 4 weeks after AAV8 injection, mice underwent either 1MNA treatment or induction of the AP model [31].

### 4.11 PF4 Neutralization

To neutralize PF4, mice were treated with one dose of PF4 neutralizing antibody (10 µg per mice, R&D System, Cat.MAB595) or the rat IgG2a isotype (control group), caerulein injection was then started 30 min after the antibody injection [71].

### 4.12 BX471 Treatment

To block CCR1, BX471 was dissolved in DMSO at a concertation of 20 mg/mL and administered subcutaneously to mice at a dose of 100 mg/kg. Caerulein injections were initiated 30 min after BX471 injection [72].

### 4.13 Histopathological Analysis

Fresh pancreas was collected, fixed in 4% paraformaldehyde and further embedded in paraffin. Paraffin‐embedded pancreas was sectioned at a thickness of 4 µm, and then stained with hematoxylin and eosin (H&E). The pancreatic injury score was calculated as described [19, 73]. At least three fields of each sample were randomly captured. The severity of pancreatic injury was evaluated for each parameter, including edema, inflammation, necrosis and vacuolization.

### 4.14 Biochemical Assays

The cytokines TNF‐α, IL‐1β, IL‐6, IL‐4 and IL‐10 were quantitated using ELISA kits (CUSABIO, Wuhan, China). Mice serum lipase and amylase levels were measured by commercial kits (Nanjing Jiancheng Corp, Nanjing, China) according to the manufacturer's protocols and detected by Varioskan LUX. The serum PF4 levels were determined using a corresponding ELISA kit (CUSABIO, Wuhan, China).

### 4.15 Flow Cytometry Analysis

Pancreatic immune cells were isolated using collagenase P digestion method and then separated through a 70 µm cell filter [74]. Human blood samples were mixed with Percoll and subjected to density gradient centrifugation at 800 g for 30 min to obtain single‐cell suspensions of PBMCs. After washing and resuspending of cells, 1 × 10^6^ cells/well were plated in a 24 well plate and co‐incubated with stimulation cocktail for 5 h. After cells were washed with FACS buffer and surface stained with FVD (Thermo Fisher Scientific, Cat.L34957), CD45 (BioLegend, Cat.103132), mouse CD11b (BioLegend, Cat.101226), mouse F4/80 (BioLegend, Cat.123113), mouse Ly6g (BioLegend, Cat.127607), mouse CCR1 (BioLegend, Cat.152503), mouse CD4 (BioLegend, Cat.100555), human CD3 (BioLegend, Cat.317335), human CD4 (BioLegend, Cat.300517), human CD25 (BioLegend, Cat.356103), human CD14 (BioLegend, Cat.325617), human CD16 (BioLegend, Cat.302011) antibodies at 4°C for 60 min. Subsequently, the cells were fixed and permeabilized by using the Cell Fixation & Permeabilization Kit (Thermo Fisher Scientific, 00‐5521‐00) and then stained with mouse GATA3 (BioLegend, Cat.653807), mouse PF4 (Abcam, Cat.ab303494), human FOXP3 (BioLegend, Cat.320123), human PF4 (Proteintech, Cat.98410‐3‐RR) antibodies at 4°C for 60 min. Thereafter that the second antibody anti‐rabbit IgG (BioLegend, Cat.406410) was added and incubated at 4°C for further 60 min. Finally, stained cells were carried out on BD FACS Celesta (BD Biosciences, San Jose, CA) and analyzed by Flow Jo.

### 4.16 Real‐Time Quantitative PCR Analysis

The pancreas, colon and macrophages were digested and total RNA was extracted with TRIzol reagent (Invitrogen, USA). Complementary DNA was generated by reverse transcription using the PrimeScript RT Reagent Kit (Takara, Japan). Real‐time qPCR was carried out with the ABI 7500 real‐time PCR system (Applied Biosystems). The primers for real‐time qPCR are shown in Table S4. Total DNA was extracted from mice feces with the QIAamp DNA stool mini kit (QIAGEN). Total DNA expression was calculated using the ABI 7500 real‐time PCR system (Applied Biosystems) with a corresponding primer (Table S4).

### 4.17 Immunofluorescence Staining

The pancreatic tissue was embedded in paraffin and sectioned at 4‐µm thickness, then incubated with F4/80 (Abcam, Cat#EPR26545‐166) and ELF4 (Abmart, Cat#TD4678S) antibodies at 4°C overnight and further incubated with secondary antibodies (Beyotime, A5021, A0423) for 1 h at room temperature. The nuclei were stained with 4°C, 6‐diamidino‐2‐phenylindole (DAPI) (Beyotime, C1002) at room temperature for 1 h. Immunofluorescence images were examined by confocal microscope.

### 4.18 Surface‐Plasmon Resonance Measurement

The interaction between 1MNA and ELF4 was quantitatively measured using a Biacore Insight system (MA. USA) with a CM5 sensor chip. Purified recombinant ELF4 protein was immobilized on the CM5 chip and activated with 10 mM glycine hydrochloride solution (pH 2.0). Gradient concentrations of 1MNA (0.3125, 0.625, 1.25, 2.5, 5, 10 µmol/L) were injected as analytes. Data were analyzed using Biacore Insight dedicated software (Cytiva, V6.0 Marborough, MA.USA).

### 4.19 Western Blot Analysis

Proteins from pancreatic tissues and macrophages were extracted according to the manufacturer's instructions (Beyotime, P0133B). Western blotting was performed using primary antibodies against PF4 (Proteintech, Cat.21157‐1‐AP), ELF4 (Abmart, Cat.TP72086S) and GAPDH (Proteintech, Cat.60004‐1‐Ig), each at a dilution 1:1000. Membranes were subsequently incubated with appropriate secondary antibodies (Li‐COR, Lincoln, NE, USA). Protein expression levels were quantified using Image Studio Lite software.

### 4.20 Bone Marrow Derived Macrophages Isolation and Co‐Culture

Mice bone marrow‐derived macrophages (BMDMs) were isolated from wild‐type C57BL/6 mice or *Pf4* ^−/−^ mice. BMDMs were cultured in Dulbecco's modified Eagle's medium DMEM medium (Gibco, USA) contained 10% fetal bovine serum FBS (Sigma‐Aldrich, USA), 1% penicillin/streptomycin (Gibco, USA) and 20 ng/mL macrophage colony‐stimulating factor (M‐CSF) (PeproTech, USA) for differentiation [40, 75]. BMDMs were collected after five to seven days of culture. Pancreatic acinar cells were isolated from fresh pancreas as previously described (Huang et al., 2017) and grown in DMEM medium (Gibco, USA) supplemented with 10% FBS (Sigma‐Aldrich, USA), 1% penicillin/streptomycin (Gibco, USA) [19, 76]. Pancreatic acinar cells were stimulated with 1 µM CCK over 30 min. BMDMs were co‐incubated with activated‐acinar cells or untreated cells (control group) for 6h [40]. Subsequently, the co‐incubated cells were washed by PBS and collected.

### 4.21 Statistical Analysis

By using randomization and blinded analysis, all animal group sizes for statistical analysis were designed to be equal. The clinical group sizes were determined by previous experience, but not by statistical methods. No statistical data were excluded, and outliers were included in the statistical analysis. Data from at least three independent experiments performed in triplicates are presented as the means ± SEM. Differences between two groups were assessed using a Student's *t* test (for parametric data, and Gaussian distribution) and the nonparametric Mann–Whitney test (for nonparametric data, and skewed distribution). Statistical analysis for three or more groups was performed by one‐way analysis of variance (ANOVA) (for parametric data, and Gaussian distribution) and nonparametric Kruskal Wallis test (for nonparametric data, and skewed distribution). The method for multiple testing correction is the Tukey post hoc test. Bray‐Curtis dissimilarity matrices were calculated and used for ordination by PCoA. All correlation analysis was tested with Spearman's correlation. Statistical analysis was performed using SPSS v.21.0 (IBM) or Prism v.8.0 (GraphPad Software). Statistical significance was defined as follows: *p < 0.05, *p < 0.01 and *p < 0.001. Graphical abstract was generated using BioRender.

## Author Contributions

L.W.L., Q.G.L., L.L. and B.S. conceptualized and designed the study. D.X.L., H.R.D., T.Q.L., Y.H.S., R.K., H.C., X.W.B., H.T.T., D.B.X. and X.Z.M. participated in collection, preparation, interpretation, validation and critical review of the data. G.Q.L., T.Q.L., Y.H.S., C.Z. and Y.X. performed the cell isolation, culture, and detection experiments. L.W.L., G.Q.L., L.L., D.X.L., H.R.D. and Y.H.S. conducted animal experiments. L.W.L. and G.Q.L. performed histological evaluation. L.W.L. and G.Q.L. performed bioinformatics analysis and statistical analysis. L.W.L. created the figures, tables and supplementary materials. L.W.L. and L.L. drafted the manuscript. B.S. reviewed and edited the manuscript text. L.W.L., Q.G.L., L.L. and B.S. acquired funding for the study.

## Conflicts of Interest

The authors declare no conflicts of interest.

## Supporting information




**Supporting File 1**: advs75823‐sup‐0001‐SuppMat.docx.

**Supporting File 2**: advs75823‐sup‐0002‐TableS1.docx.

**Supporting File 3**:: advs75823‐sup‐0003‐TableS2.docx.

**Supporting File 4**: advs75823‐sup‐0004‐TableS3.docx.

**Supporting File 5**: advs75823‐sup‐0005‐TableS4.docx.

## Data Availability

The data that support the findings of this study are available from the corresponding author upon reasonable request.
