# Supplementary material for: Gut Microbiota Regulates Systemic Inflammatory Response and Compensatory Anti‐Inflammatory Response Syndromes by Targeting PF4+ Macrophages in Acute Pancreatitis
Source: Adv Sci (Weinh). 2026 May 26;13(46):e11193. doi: 10.1002/advs.202511193 (PMC13335921; doi:10.1002/advs.202511193)
Supplement: Supplementary file 2 [file ADVS-13-e11193-s002.docx]

**Gut microbiota regulates systemic inflammatory response and compensatory anti-inflammatory response syndromes by targeting PF4^+^macrophages in acute pancreatitis**

Liwei Liu^1,2#^, Guanqun Li^2,3#^, Dongxu Lu^1,2^, Haoran Ding^1,2^, Tianqi Lu^2,3^, Yuhang Sui^2,3^, Can Zhang^2,3^, Yu Xie^2^, Rui Kong^2,3^, Hua Chen^2,3^, Xuewei Bai^2,3^, Hongtao Tan^2,3^, Dongbo Xue^1,2^, Xianzhi Meng^1,2^, Le Li^3*^, Bei Sun^2,3*^

1. Department of Minimally Invasive Biliary Surgery, The First Affiliated Hospital of Harbin Medical University, Harbin 150001, China.

2. Key Laboratory of Hepatosplenic Surgery, Ministry of Education, Harbin 150001, China.

3. Department of Pancreatic and Biliary Surgery, The First Affiliated Hospital of Harbin Medical

University, Harbin 150001, China.

^#^ Liwei Liu and Guanqun Li are equally contributed to this study.

* Corresponding authors

Bei Sun

Department of Pancreatic and Biliary Surgery

The First Affiliated Hospital of Harbin Medical University

Harbin, China, 150081

sunbei70@hrbmu.edu.cn

Le Li

Department of Pancreatic and Biliary Surgery

The First Affiliated Hospital of Harbin Medical University

Harbin, China, 150081

lile@hrbmu.edu.cn

**Supplementary Fig. 1** **The correlation analysis between gut microbiota and immune profiles in AP patients. a.** Relative abundance of the top 10 most abundant gut microbiota in the phylum level. **b.** Top 15 average abundance of gut microbiota were compared among HC, MAP and SAP group. **c.** Relative abundance of the top 15 gut microbiota at the genus level. **d.** Relative abundance of the genera *Enterococcus* in each sample. The ROC curve discriminating SAP vs MAP using the abundance of *Enterococcus* in patients. **e.** The proportion of the total T cells. **f.** Dot plot of each cell cluster depicting the percentages and average expressions of the canonical genes. **g.** The relative expression level of *CD14* (marker gene of monocytes) was detected in hPBMCs using qPCR. **h.** The relative expression level of *GATA3* (marker gene of Th2 cells) was detected in hPBMCs using qPCR. **i.** The relative expression level of *CD8a* (marker gene of CD8+T cells) was detected in hPBMCs using qPCR. **j.** Enriched biological processes by differentially expressed genes in classical monocytes between SAP patients and MAP patients using GO analysis. **k.** Correlation analysis between the abundance of *Bacteroides* and the prevalence of macrophages (95% confidence interval: -0.8236 to -0.08069). **l.** Correlation analysis between the abundance of *Bacteroides* and prevalence Th2 cells (95% confidence interval: -0.8689 to -0.2368). *P* values were determined by two-tailed ordinary one-way ANOVA with the Tukey post hoc test or Student’s *t*-test. Data was represented as mean ± SEM. **p* < 0.05.

**Supplementary Fig. 2 Gut microbiota dysbiosis triggers the SIRS/CARS phenotype in AP mice.** The FMT experiment was divided into four groups (ABX, n =6; FMT-HC, n = 9; FMT-MAP, n = 9; FMT-SAP, n = 9). **a.** Serum IL-10 levels in each sample. **b.** Flow cytometric quantiﬁcation indicating the percentage of macrophages, neutrophils and CD4+T cells in the pancreas (n = 6). **c.** A UMAP displaying eight macrophage subclusters obtained from FMT-SAP (n = 3) and FMT-HC (n = 3) mice. **d.** Top three marker genes in each cell subcluster. **e.** GSEA snapshots of cytokine-cytokine receptor interaction pathway between FMT-SAP mice and FMT-HC mice. **f.** Enriched pathways predicted by differentially expressed genes in C0 macrophage cluster between FMT-SAP and FMT-HC mice using KEGG analysis. **g.** The *Pf4* gene expression was detected in the pancreas of FMT-treated mice using qPCR. *P* values were determined by two-tailed ordinary one-way ANOVA with the Tukey post hoc test or Student’s *t*-test. Data was represented as mean ± SEM. **p* < 0.05.

**Supplementary Fig. 3 PF4^+^macrophages promote SIRS/SARS phenotypes in SAP mice. a.** Serum lipase levels in *Pf4*^-/-^ mice (n = 5). **b-c.** Serum amylase and lipase levels in PF4-neutralized mice (n = 5). **d-e.** Serum IL-4 and IL-1β levels (n = 6). **f.** Representative Hematoxylin and eosin (H&E) staining of pancreatic tissues from *Pf4*^-/-^mice the duct ligation + caerulein (CL) -SAP model mice (scale bar = 100 μm) and corresponding hepatological scores (n = 6). **g-h.** Serum amylase and lipase levels (n = 6). **i-k.** Flow cytometric quantiﬁcation of infiltrating PF4^+^macrophages, CCR1^+^neutrophils and CCR1^+^Th2 cells in the pancreas of *Pf4*^-/-^ SAP mice (n = 5). **l-m.** Flow cytometric analysis of pancreatic macrophages infiltration in AP mice treated with clod-lipo (n = 6). **n-o.** Serum IL-10 and IL-1β levels in the serum of BMDMs-transferred mice (n = 6). **p-q.** Immunohistochemical quantiﬁcation and statistical analysis of pancreatic macrophages infiltration in *Pf4*^-/-^ mice treated with clod-lipo (n = 5). **r-v.** Adoptive-transfer of BMDMs from wild-type mice and *Pf4*^-/-^ mice into clod-lipo-treated *Pf4*^-/-^mice. **r.** Pancreatic histological scores (n = 6). **s.** Representative H&E staining of pancreatic tissues (scale bar = 100 μm, n = 6). **t-v.** Serum amylase, lipase and IL-1β levels (n = 6). *P* values were determined by two-tailed Student’s *t*-test. Data was represented as mean ± SEM. **p* < 0.05.

**Supplementary Fig. 4 PF4^+^macrophages activate innate and adaptive immune response in AP mice. a.** GO analysis of enriched biological processes based on differentially expressed genes between the C0 cluster and C1-7 clusters. **b.** UMAP illustrating interactions between macrophages and neutrophils, and between macrophages and T cells. **c.** Proportion of neutrophils in FMT-SAP and FMT-HC mice determined by scRNA-seq. **d-e.** scRNA-seq analysis of *Cd4* and *Gata3* expressions in immune cells. **f-g.** scRNA-seq analysis of *Ccr1* expression in neutrophils cells and Th2 cells. **h-p.** AP mice pretreated with CCR1 inhibitor BX471 (n = 6). **h.** Experimental scheme for BX471 pretreatment experiment (n = 6). **i-j.** Representative H&E staining images and histological score quantification of pancreatic tissues from BX471-treated mice (scale bar = 100 μm, n = 6). **k-m.** Serum lipase, amylase and IL-1β levels (n = 6). **n-p.** Flow cytometric quantiﬁcation of infiltrating CCR1^+^neutrophils, CCR1^+^Th2 cells and PF4^+^macrophages in the pancreas of BX471-pretreated AP mice (n = 6). *P* values were determined by two-tailed Student’s *t*-test. Data was represented as mean ± SEM. **p* < 0.05.

**Supplementary Fig. 5 *B.thetaiotaomicron* suppresses immune disorder-mediated SIRS/CARS phenotype in AP mice. a.** Relative abundance of *B.thetaiotaomicron* in fecal samples from AP mice treated with *B.thetaiotaomicron* (n = 7). **b.** Representative western blot images of ELF4 and PF4 protein expressions in the pancreas of *B.thetaiotaomicron*-treated mice (n = 6), original blots were found in Supplementary Fig. 12c. **c-d.** Quantification of ELF4 and PF4 protein levels in the pancreas (n = 6). **e.** Schematic representation of the *B.thetaiotaomicron* supplementation experiment. WT mice were pre-treated with antibiotics and *B.thetaiotaomicron*, and then subjected to the construction of the duct ligation + caerulein (CL)-SAP model and sodium taurocholate (Tau)-SAP model. **f-g.** Representative H&E staining images and histological score quantification of pancreatic tissues from *B.thetaiotaomicron*-treated mice (scale bar = 100 μm, n = 6). **h-k.** Flow cytometric quantiﬁcation of infiltrating PF4^+^macrophages, CCR1^+^neutrophils and CCR1^+^Th2 cells in the pancreas of *B.thetaiotaomicron*-treated vs PBS-treated CL-SAP mice (n = 6). **l-m.** Representative H&E staining images and histological score quantification of pancreatic tissues from *B.thetaiotaomicron*-treated Tau-SAP mice and PBS-treated Tau-SAP mice (scale bar = 100 μm, n = 5). **n-q.** Flow cytometric quantiﬁcation and statistical analysis of infiltrating PF4^+^macrophages, CCR1^+^neutrophils and CCR1^+^Th2 cells in the pancreas of *B.thetaiotaomicron*-treated vs PBS-treated Tau-SAP mice (n = 5). *P* values were determined by two-tailed Student’s *t*-test. Data was represented as mean ± SEM. **p* < 0.05.

**Supplementary Fig. 6 *B.thetaiotaomicron*-derived intestinal 1MNA alleviates SIRS/CARS phenotype in AP mice. a.** Correlation analysis between the abundance of the top 10 bacterial genera and serum metabolites in FMT-treated mice. **b.** Schematic illustration of the 1MNA biosynthesis pathway. **c.** NCBI analysis showing sequence coincidence *nada* and *metk* genes in *B.thetaiotaomicron*. **d.** Elisa measure of pancreatic 1MNA levels in FMT-treated mice (n = 9). **e.** SAM levels in the colon of FMT-treated mice (n = 6). **f-g.** qPCR analysis of *Nnmt* gene expression in the pancreas of FMT-treated mice and *B.thetaiotaomicron*-treated mice. **h-i.** Serum amylase and lipase levels (n = 5). **j-l.** Serum IL-1β, IL-10 and IL-4 levels in each sample (n = 5). **m-n.** Flow cytometric quantification of CCR1^+^neutrophils and CCR1^+^Th2 cells in the pancreas (n = 5). *P* values were determined by two-tailed ordinary one-way ANOVA with the Tukey post hoc test or Student’s *t*-test. Data was represented as mean ± SEM. **p* < 0.05.

**Supplementary Fig. 7 1MNA ameliorates SIRS/CARS phenotype in SAP mice. a.** Schematic illustration of experimental design. CL-SAP mice were pre-treated with 1MNA and then randomly assigned to CL-SAP group and 1MNA+CL-SAP group. **b-c.** Representative H&E staining images and histological score quantiﬁcation from 1MNA-treated mice (scale bar = 100 μm, n = 6). **d-g.** Flow cytometric quantiﬁcation and statistical analysis of infiltrating PF4^+^macrophages, CCR1^+^neutrophils and CCR1^+^Th2 cells in the pancreas of 1MNA-treated vs PBS-treated CL-SAP mice (n = 6). **h.** Schematic illustration of experimental design. WT mice and *Pf4*^-/-^mice were pre-treated with 1MNA and sodium taurocholate, and then randomly assigned to Tau-SAP group, Tau-SAP+1MNA group and Tau-SAP+*Pf4*^-/-^ group. **i-j.** Representative H&E staining images and histological score quantiﬁcation from 1MNA-treated mice and PF4 deficiency (scale bar = 100 μm, n = 5). **k.** Flow cytometric statistical analysis indicating the percentage of PF4^+^macrophages in the pancreas (n = 5). **l.** Flow cytometric quantiﬁcation of infiltrating PF4^+^macrophages, CCR1^+^neutrophils and CCR1^+^Th2 cells in each sample (n = 5). **m.** Flow cytometric statistical analysis indicating the percentage of CCR1^+^neutrophils in the pancreas (n = 5). **n.** Flow cytometric statistical analysis indicating the percentage of CCR1^+^Th2 cells in the pancreas (n = 5). *P* values were determined by two-tailed Student’s *t*-test. Data was represented as mean ± SEM. **p* < 0.05.

**Supplementary Fig. 8 *B.thetaiotaomicron*-derived intestinal 1MNA inhibits PF4 in macrophages by targeting ELF4. a.** Intersection analysis between differentially expressed genes in macrophages and eight known transcriptional factors. **b-d.** Representative western blot images and quantification of ELF4 and PF4 protein levels in AP mice treated with 1MNA (n = 6), original blots were found in Supplementary Fig. 12b. **e-f.** Relative mRNA expression of *Pf4* and *Elf4* in BMDMs treated with PBS or 1MNA (n = 5). **g.** Luciferase reporter assay showing ELF4-mediated transcriptional activity of the *Pf4* promoter *in vitro* (n = 3). **h-i.** Molecular dynamics simulation illustrating the interaction between 1MNA and ELF4 protein. **j-k.** Representative western blot quantification analysis of ELF4 and PF4 protein levels in macrophages treated with si-ELF4 and 1MNA (n = 5). **l-m.** Relative mRNA expressions of *Pf4* and *Elf4* in macrophages treated with si-ELF4 and 1MNA (n = 5). *P* values were determined by two-tailed ordinary one-way ANOVA with the Tukey post hoc test or Student’s *t*-test. Data was represented as mean ± SEM. **p* < 0.05.

**Supplementary Fig. 9 *B.thetaiotaomicron*-derived intestinal 1MNA alleviates immune disorders-exacerbated AP injury by targeting ELF4 in macrophages. a-j.** *Elf4* cDNA was cloned into the single-stranded AAV8 vector; the AAV8 empty vector was used as a control (AAV-NC). WT mice were pretreated with AAV-*MacElf*4, and then administered 1MNA and underwent SAP model after 4 weeks. These mice were randomly assigned to CL-SAP+AAV-NC group, CL-SAP+1MNA+AAV-NC group, CL-SAP+AAV-*MacElf4* group and CL-SAP+1MNA+AAV-*MacElf4* group. **a.** Immunofluorescence double staining of ELF4 (green) and F4/80 (red) of ELF4^+^macrophages in the pancreas (Scale bar= 20µm, n = 5). **b-c.** Representative H&E staining images and histological score quantiﬁcation of pancreatic injury (scale bar = 100 μm, n = 5). **d.** Serum amylase levels in each sample (n = 5). **e.** Serum TNF-a levels in each sample (n = 5). **f.** Serum IL-4 levels in each sample (n = 5). **g.** Flow cytometric statistical analysis indicating the percentage of PF4^+^macrophages in the pancreas (n = 5). **h.** Flow cytometric quantiﬁcation of infiltrated PF4^+^macrophages, CCR1^+^neutrophils and CCR1^+^Th2 cells inﬁltration in each sample (n = 5). **i.** Flow cytometric statistical analysis indicating the percentage of CCR1^+^neutrophils in the pancreas (n = 5). **j.** Flow cytometric statistical analysis indicating the percentage of CCR1^+^Th2 cells in the pancreas (n = 5). *P* values were determined by two-tailed ordinary one-way ANOVA with the Tukey post hoc test or Student’s *t*-test. Data was represented as mean ± SEM. **p* < 0.05.

**Supplementary Fig. 10 PF4^+^macrophages activate immune response in AP patients.** Human feces and blood samples were collected from two independent cohorts, including cohort 1 (year, 2020-2022; HC, n =29; MAP, n = 43; SAP, n = 20) and cohort 2 (year, 2023-2026; MAP, n = 36; SAP, n = 26). **a.** Violin plots indicating the expressions of marker genes in each monocyte cluster from hPBMCs. **b.** GO analysis of enriched biological processes predicted from differentially expressed genes in C7 macrophage cluster between SAP and MAP patients. **c.** Serum PF4 levels of each human sample in cohort 1. **d.** The ROC curve distinguishing SAP vs MAP patients in cohort 1 using PF4 expression. **e.** Serum PF4 levels of each human sample in cohort 2. **f.** The ROC curve distinguishing SAP vs MAP patients in cohort 2 using PF4 expression. **g.** Flow cytometric statistical analysis of PF4^+^macrophages in peripheral blood from cohort 1. **h.** The ROC curve distinguishing SAP vs MAP patients in cohort 1 using PF4^+^macrophages proportion expression. **i.** qPCR statistical analysis of *B.thetaiotaomicron* abundance in cohort 2. **j.** The ROC curve distinguishing SAP vs MAP patients in cohort 2 using the abundance of *B.thetaiotaomicron*. **k.** Correlation analysis between the serum 1MNA levels and the proportion of PF4^+^macrophages in AP patients from cohort 1 (95% confidence interval: 0.1882 to 0.6049). **l.** Correlation analysis between the abundance of *B.thetaiotaomicron* and the serum PF4 levels in AP patients from cohort 1 (95% confidence interval: -0.5313 to -0.02022). **m.** Correlation analysis between the abundance of *B.thetaiotaomicron* and the serum PF4 levels in AP patients from cohort 2 (95% confidence interval: -0.5725 to -0.1399). **n.** Flow cytometric statistical analysis of Treg cells in peripheral blood from cohort 1. **o.** Correlation analysis between the proportion of PF4^+^macrophages, the proportion of Treg cells, and clinical index in cohort 1. *P* values were determined by two-tailed ordinary one-way ANOVA with the Tukey post hoc test or Student’s *t*-test. All correlation analysis was tested with Spearman’s correlation. Data was represented as mean ± SEM. **p* < 0.05.

**Supplementary Fig. 11 Flow cytometric Gating strategy in AP patients and AP mice.** **a.** Gating strategy for PF4^+^macrophages, CCR1^+^neutrophils and CCR1^+^Th2 cells from AP mice. **b.** Gating strategy for macrophages from AP patients.

**Supplementary Fig. 12 Western blot images of ELF4 and PF4 proteins.** **a.** Original western blot images of ELF4 and PF4 proteins in macrophages treated with si-ELF4 and 1MNA. **b.** Original western blot images of ELF4 and PF4 protein levels in AP mice treated with 1MNA. **c.** Original western blot images of ELF4 and PF4 protein levels in AP mice treated with *B.thetaiotaomicron*.
