# Supplementary material for: Gut Microbiota Regulates Systemic Inflammatory Response and Compensatory Anti‐Inflammatory Response Syndromes by Targeting PF4+ Macrophages in Acute Pancreatitis
Source: Adv Sci (Weinh). 2026 May 26;13(46):e11193. doi: 10.1002/advs.202511193 (PMC13335921; doi:10.1002/advs.202511193)
Supplement: Supplementary file 3 [file ADVS-13-e11193-s003.docx]

**Supplementary Table 2 Demographic and clinical characteristics of the second cohort**

| **Characteristics** | **MAP(n=36)** | **SAP(n=26)** | **p value** |
| --- | --- | --- | --- |
| Gender,Male,n,(%)^#^ | 17(47.2%) | 8(30.8%) | 0.294 |
| Age(years), mean(SD)* | 48.67(±16.47) | 50.92(±12.27) | 0.558 |
| BMI,mean(SD)* | 25.73(±4.30) | 24.74(±3.99) | 0.358 |
| Drinking, yes,n,(%)^#^ | 6(16.7%) | 4(15.4%) | 1.000 |
| Smoking, yes,n,(%)^#^ | 5(13.9%) | 4(15.4%) | 1.000 |
| Hypertension, yes,n,(%)^#^ | 6(16.7%) | 6(23.1%) | 0.536 |
| Diabetes, yes,n,(%)^#^ | 10(27.8%) | 6(23.1%) | 0.773 |
| IPN, yes,n,(%)^#^ | 1(2.8%) | 19(73.1%) | ＜0.0001 |
| Hemoglobin, mean(SD)* | 127.89(±21.25) | 113.85(±37.36) | 0.065 |
| AST, mean(SD)* | 39.98(±52.14) | 31.98(±49.32) | 0.545 |
| ALT, mean(SD)* | 48.30(±60.76) | 32.66(±30.79) | 0.233 |
| Bilirubin, mean(SD)* | 32.13(±66.48) | 21.61(±36.16) | 0.467 |
| Albumin, mean(SD)* | 35.25(±6.72) | 32.98(±8.71) | 0.252 |
| Creatinine, mean(SD)* | 6.20(±8.85) | 26.69(±42.80) | 0.007 |
| Urea_nitrogen, mean(SD)* | 67.77(±43.02) | 70.29(±54.54) | 0.840 |
| CRP, mean(SD)* | 64.35(±87.91) | 149.46(±117.93) | 0.002 |
| PCT, mean(SD)* | 9.05(±38.71) | 1.97(±3.86) | 0.357 |
| ICU, yes,n,(%)^#^ | 2(5.6%) | 10(38.5%) | 0.002 |
| LPN+PCD, yes,n,(%)^#^ | 2(5.6%) | 22(84.6%) | ＜0.0001 |

*Independent two-sample t-test; ^#^Two-sample z-test for proportions.
