# Supplementary material for: Gut Microbiota Regulates Systemic Inflammatory Response and Compensatory Anti‐Inflammatory Response Syndromes by Targeting PF4+ Macrophages in Acute Pancreatitis
Source: Adv Sci (Weinh). 2026 May 26;13(46):e11193. doi: 10.1002/advs.202511193 (PMC13335921; doi:10.1002/advs.202511193)
Supplement: Supplementary file 4 [file ADVS-13-e11193-s004.docx]

| Characteristics | HC(n=3) | AP(n=3) | SAP(n=3) | p value |
| --- | --- | --- | --- | --- |
| Male, n (%)^*^ | 2(66.67) | 2(66.67) | 2(66.67) | 1 |
| Age(years), mean (SD)^#^ | 52.67(±6.66) | 50.33(±14.5） | 58.00(±15.72） | 0.767 |
| Weight(kg), mean (SD)^#^ | 89.00(±11.53) | 75.77(±4.25） | 80.67(±19.14） | 0.50 |

**Supplementary Table 3 Demographic and clinical characteristics of healthy controls and AP patients**

*Fisher's exact test; ^#^Two-tailed ordinary one-way ANOVA.
