# Supplementary material for: Gut Microbiota Regulates Systemic Inflammatory Response and Compensatory Anti‐Inflammatory Response Syndromes by Targeting PF4+ Macrophages in Acute Pancreatitis
Source: Adv Sci (Weinh). 2026 May 26:e11193. Online ahead of print. doi: 10.1002/advs.202511193 (PMC13335921; doi:10.1002/advs.202511193)
Supplement: Supplementary file 2 — Supporting File 2: advs75823‐sup‐0002‐TableS1.docx. [file ADVS-9999-e11193-s002.docx]

**Supplementary Table 1 Demographic and clinical characteristics of the first cohort**

| **Characteristics** | **MAP(n=43)** | **SAP(n=20)** | **p value** |
| --- | --- | --- | --- |
| Gender,Male,n,(%)^#^ | 25(58.1%) | 15(75.0%) | 0.196 |
| Age(years), mean(SD)* | 48.97(±15.80) | 45.90(±12.85) | 0.450 |
| BMI,mean(SD)* | 26.97(±5.43) | 23.72(±4.05) | 0.021 |
| Drinking, yes,n,(%)^#^ | 13(30.2%) | 5(25.0%) | 0.669 |
| Smoking, yes,n,(%)^#^ | 5(13.9%) | 4(15.4%) | 1.000 |
| Hypertension, yes,n,(%)^#^ | 11(25.6%) | 5(25.0%) | 0.961 |
| Diabetes, yes,n,(%)^#^ | 12(27.9%) | 5(25.0%) | 0.809 |
| IPN, yes,n,(%)^#^ | 6(14.0%) | 10(50.0%) | 0.002 |
| Hemoglobin, mean(SD)* | 150.96(±77.11) | 115.05(±35.82) | 0.052 |
| AST, mean(SD)* | 74.14(147.12) | 86.74(±130.83) | 0.745 |
| ALT, mean(SD)* | 66.79(±99.48) | 67.58(±131.13) | 0.979 |
| Bilirubin, mean(SD)* | 47.17(±117.21) | 23.41(±18.59) | 0.373 |
| Albumin, mean(SD)* | 37.19(±6.09) | 33.52(±5.04) | 0.202 |
| Creatinine, mean(SD)* | 65.62(±30.58) | 83.79(±57.67) | 0.107 |
| Urea_nitrogen, mean(SD)* | 9.60(±27.64) | 6.52(±4.99) | 0.624 |
| CRP, mean(SD)* | 141.38(±114.17) | 120.17(±100.87) | 0.480 |
| PCT, mean(SD)* | 3.36(±15.27) | 0.65(±0.89) | 0.432 |
| ICU, yes,n,(%)^#^ | 0(0.0%) | 5(25.0%) | 0.001 |
| LPN+PCD, yes,n,(%)^#^ | 4(9.3%) | 10(50.0%) | 0.001 |

*Independent two-sample t-test; # Two-sample z-test for proportions.
