# Supplementary material for: Gut Microbiota Regulates Systemic Inflammatory Response and Compensatory Anti‐Inflammatory Response Syndromes by Targeting PF4+ Macrophages in Acute Pancreatitis
Source: Adv Sci (Weinh). 2026 May 26:e11193. Online ahead of print. doi: 10.1002/advs.202511193 (PMC13335921; doi:10.1002/advs.202511193)
Supplement: Supplementary file 5 — Supporting File 5: advs75823‐sup‐0005‐TableS4.docx. [file ADVS-9999-e11193-s004.docx]

| Target genes | Sequences |
| --- | --- |
| *Pf4* mmu Forward 5’-GTTGTTTCTGCCAGCGGTGGTT-3′  Reverse 5’-ACAGTGGCGTCCTGCCTTGATC-3′  *Elf4* mmu Forward 5’-ATGCTTGCCAGCCCACTACAGA-3′  Reverse 5’-CCATTGGTCAGCACCGTAGTCA-3′  *Nnmt* mmu Forward 5’-GGGACCTGAGAAGGAGGAGAA-3’  Reverse 5’-CAGTCAGCAGGAGGCAGAGA-3’  *Gapdh* mmu Forward 5-CATCACTGCCACCCAGAAGACTG-3  Reverse 5-ATGCCAGTGAGCTTCCCGTTCAG-3  *PF4* hsa Forward 5’-TCCTGCCACTTGTGGTCGCCT-3′  Reverse 5’-CCTTGATCACCTCCAGGCTGG-3′  *ELF4* hsa Forward 5’-AATTGGGACCGTCGCTAGACGA-3′  Reverse 5’-GTGGATGTTGCTGGGCACTGAA-3′  *CD14* hsa Forward 5’-CTGGAACAGGTGCCTAAAGGAC-3′  Reverse 5’-GTCCAGTGTCAGGTTATCCACC-3′  *GATA3* hsa Forward 5’-ACCACAACCACACTCTGGAGGA-3′  Reverse 5’-TCGGTTTCTGGTCTGGATGCCT-3′  *β-Actin* has Forward 5′-GTGGGGCGCCCCAGGCACCA-3′  Reverse 5′-CTCCTTAATGTCACGCACGATTTC-3′  *CD8A* hsa Forward 5’-ACTTGTGGGGTCCTTCTCCTGT-3′  Reverse 5’-TGTCTCCCGATTTGACCACAGG-3′  *B.thetaiotaomicron* Forward 5’-CACAACAGCCATAGCGTTCCA-3′  Reverse 5’-ATCGCAAAAATAAGATGGGCAAA-3′ | |
